# Acinetobacter legionensis sp. nov. and Acinetobacter psychrotolerans sp. nov. isolated from different types of retail meat

**DOI:** 10.1099/ijsem.0.007285

**Published:** 2026-09-22

**Authors:** Alba Puente, Cristina Galisteo, José F. Cobo-Díaz, Filipa Grosso, Coral Barcenilla, Mercedes López, Miguel Prieto, Luisa Peixe, Avelino Alvarez-Ordóñez

**Affiliations:** 1 Department of Food Science and Technology, Universidad de León, León, Spain; 2 Institute of Food Science and Technology (ICTAL), Universidad de León, León, Spain; 3 Department of Animal Health, Universidad de León, León, Spain; 4 Instituto de Investigación Biosanitaria de León (IBIOLEÓN), León, Spain; 5 Department of Biological Sciences, UCIBIO–Applied Molecular Biosciences Unit, Faculty of Pharmacy, Laboratory of Microbiology, University of Porto, Porto, Portugal; 6 Associate Laboratory i4HB, Institute for Health and Bioeconomy, Faculty of Pharmacy, University of Porto, Porto, Portugal

**Keywords:** *Acinetobacter*, meat, new species, phylogenomic

## Abstract

Twelve isolates representing two novel *Acinetobacter* species were isolated from various fresh meat and meat preparation samples collected from supermarkets in *León*, Spain. These isolates were identified by matrix-assisted laser desorption/ionization time-of-flight mass spectrometry and GTDB-Tk software as members of the genus *Acinetobacter*. Phylogenomic analysis based on the core genome showed that these isolates formed two separate branches (1 including 11 isolates: ULE_I046^T^, ULE_I001, ULE_I024, ULE_I037, ULE_I053, ULE_I057, ULE_I064, ULE_I068, ULE_I075, ULE_I080 and ULE_I092; and the other only one, ULE_I010^T^) separated from all known *Acinetobacter* species. Average nucleotide identity and digital DNA–DNA hybridization values of all isolates with closely related type strains of species of the genus *Acinetobacter* supported their status as new species. The names *Acinetobacter legionensis* sp. nov. (comprising 11 isolates) and *Acinetobacter psychrotolerans* sp. nov. (comprising one strain) were proposed for the two novel taxa. Additional phylogenomic and phenotypic analyses showed that strain LMG 10606 (=RUH 2236), originally assigned to *Acinetobacter guillouiae,* belongs to *A. legionensis* sp. nov. Other two strains from the original description of *A. guillouiae* (LUH 5606 and LUH 6980) may also represent members of *A. legionensis* sp. nov., as indicated by a *rpoB* gene phylogenetic tree analysis. The phenotypic characteristics most useful for the differentiation of the novel species described in the present study from known *Acinetobacter* species were the assimilation of malonate and the inability to grow on histamine and phenylacetate of *A. legionensis* sp. nov., and the inability of *A. psychrotolerans* sp. nov. to grow over 30 °C. The type strain *A. legionensis* ULE_I046^T^ (=CECT 31169^T^=CCP 535^T^) has a genome size of 4.58 Mb and a G+C content of 37.7 mol%. The type strain *A. psychrotolerans* ULE_I010^T^ (=CECT 31168^T^=CCP 534^T^) has a genome size of 3.56 Mb, with a G+C content of 37.2 mol%.

## Introduction

 The genus *Acinetobacter*, a member of the *Moraxellaceae* family, was first described in 1954 [1] and it includes Gram-negative, non-motile, catalase-positive, oxidase-negative and aerobic bacteria. Currently, the genus comprises 90 species with validly published names, in addition to several other species without validly published names (https://lpsn.dsmz.de/genus/acinetobacter, last accessed on 23 March 2026) [2]. *Acinetobacter* species have been reported from a wide variety of habitats, including environmental sources and clinical samples. Furthermore, some species have been isolated from food products, such as milk, fruits, vegetables, fish and meat [3–6]. The current study characterizes and describes two novel species of the genus: *Acinetobacter legionensis* sp. nov. and *Acinetobacter psychrotolerans* sp. nov. Twelve isolates constituting these new species (eleven of *A. legionensis* sp. nov. and one of *A. psychrotolerans* sp. nov.) were isolated from various types of fresh meat and meat preparations from supermarkets in *León*, Spain, during a previous study focused on assessing the occurrence of *Acinetobacter* in meat [7]. However, this is not the first occasion on which *A. legionensis* sp. nov. isolates have been recovered. In 2010, Nemec *et al*. [8] proposed the name *Acinetobacter guillouiae* sp. nov. for the *Acinetobacter* genospecies (genomic species) 11 described by Bouvet and Grimont in 1986 [9]. This species consisted of 17 strains with different origins, including human and environmental sources [8]. The analysis of the *rpoB* gene sequences carried out during the characterization of the new *A. legionensis* sp. nov. showed that 3 out of those 17 strains could actually belong to the novel species *A. legionensis* reported in the current study. The new taxonomic status of one of these strains, RUH 2236 (=LMG 10606), is here confirmed through phylogenomic and phenotypic characterizations.

## Isolation

Each of the 12 isolates investigated in this study was isolated from different fresh meat and meat preparation samples collected from supermarkets in *León*, Spain (Table 1). The samples were processed as previously described by Puente *et al*. [7]. The isolates ULE_I046^T^, ULE_I001, ULE_I024, ULE_I037, ULE_I053, ULE_I057, ULE_I064, ULE_I068, ULE_I075, ULE_I080 and ULE_I092 were isolated by direct plating on CHROMagar^™^ *Acinetobacter* medium (CHROMagar, Paris, France) of a suspension obtained by adding 25 g of meat to 225 ml of Buffer Peptone Water (Merck KGaA, Darmstadt, Germany) supplemented with 1 mg l^−1^ vancomycin, 1.5 mg l^−1^ cefsulodin and 5 mg l^−1^ cephradine (Sigma-Aldrich, Saint Louis, USA), followed by the incubation of agar plates at 35 °C for 24 h. The isolate ULE_I010^T^ was collected by direct plating on the same selective medium after a 10 day incubation at 5 °C. The twelve isolates were identified by matrix-assisted laser desorption/ionization time-of-flight mass spectrometry (MALDI-TOF MS, Microflex LT model, Bruker-Daltonics, USA) as members of the genus *Acinetobacter* (with log score values >1.70). Isolates ULE_I046^T^, ULE_I001, ULE_I024, ULE_I037, ULE_I053, ULE_I057, ULE_I064, ULE_I068, ULE_I075, ULE_I080 and ULE_I092 were initially associated with *A. guillouiae*, with log score values ranging from 1.88 to 2.40, while the ULE_I010^T^ isolate was associated with *Acinetobacter johnsonii*, with a log score of 1.76. It is important to highlight that log score values ≥2.3 indicate a highly probable species identification; 2.29–2.0 indicate probable species identification; and 1.99–1.7 indicate a genus-level identification [10].

**Table 1.** Fresh meat and meat preparation samples from which the isolates of *A. legionensis* sp. nov. and *A. psychrotolerans* sp. nov. were collected.

Isolate	Sample	Product type	Date of isolation
*A. legionensis* ULE_I046^T^	Chicken meat	Mini burger meat packaged under MAP	March 2022
*A. legionensis* ULE_I001	Pork meat	Unpackaged loin chop	February 2022
*A. legionensis* ULE_I024	Pork meat	Loin packaged under vacuum	February 2022
*A. legionensis* ULE_I037	Pork meat	Unpackaged ribs	March 2022
*A. legionensis* ULE_I053	Pork meat	Ox cheek packaged under vacuum	March 2022
*A. legionensis* ULE_I057	Chicken meat	Burger meat packaged under MAP	March 2022
*A. legionensis* ULE_I064	Pork meat	Unpackaged ribs	April 2022
*A. legionensis* ULE_I068	Pork meat	Unpackaged chopped meat	April 2022
*A. legionensis* ULE_I075	Pork meat	Unpackaged loin steak	April 2022
*A. legionensis* ULE_I080	Pork meat	Unpackaged belly	April 2022
*A. legionensis* ULE_I092	Beef meat	Unpackaged fillet steak	May 2022
*A. psychrotolerans* ULE_I010^T^	Pork meat	Loin steak packaged under MAP	February 2022

MAP, modified atmosphere packaging.

Although the natural habitat of the new species described here is likely to be environmental, meat is very frequently contaminated by *Acinetobacter* spp. during processing and also provides favourable conditions for these bacteria to persist and multiply, as indicated by the relatively high prevalence and concentration of *Acinetobacter* spp. observed previously in fresh meat and meat preparations [7, 11, 12].

## Genome features

The genomic DNA of these isolates was extracted using the DNeasy Blood and Tissue Kit (Qiagen, Hilden, Germany) following the manufacturer’s protocol for Gram-negative bacteria with a final elution with 100 µl of Buffer AE (10 mM Tris-Cl, 0.5 mM EDTA). Sequencing libraries were obtained using the Nextera XT DNA Library Preparation kit (Illumina Inc., San Diego, CA, USA) according to the manufacturer’s instructions, and sequenced on the Illumina NovaSeq 6000 Sequencing System (Macrogen Inc., Seoul, Korea) by 150-bp PE approach. Trim Galore (v.0.6.6) (https://github.com/FelixKrueger/TrimGalore) was used for quality filtering of the reads, and contaminant DNA was eliminated using Bowtie2 (v.2.2.9) with *--sensitive-local* parameter, removing reads from the phiX174 Illumina spike-in (NCBI accession number NC_001422) as well as potential human contamination (using the GRCh38.p13 human genome, NCBI accession number GCF_000001405.39). Genomes were assembled by SPAdes (v.3.15.2) [13], and assembly quality was checked with CheckM2 [14] while Bakta Web (Software: 1.10.3, DB: 5.1.0, https://bakta.computational.bio/) was employed to detect the number of coding DNA, tRNA and rRNA sequences per genome [15]. The assembly quality statistics of the 12 isolates investigated in this study are summarized in Table S1 (available in the online Supplementary Material). Genomes were taxonomically assigned by GTDB-Tk (v.1.7.0) [16]. According to this database, the 12 isolates were identified as members of the genus *Acinetobacter* but could not be classified at the species level.

The *16S rRNA* gene sequence of the 12 isolates was extracted from their genomes by blast against a database built with *16S rRNA* gene sequences from representative *Acinetobacter* species (Table S2) employing the *-outfmt ‘6 std qseq’* command. Additionally, comparison of the *16S rRNA* gene sequences against the EzBioCloud database (https://www.ezbiocloud.net/) [17] revealed that the isolate ULE_I010^T^ had the highest sequence similarity with *Acinetobacter silvestris* ANC 4999^T^ (99.52%), and ULE_I046^T^, ULE_I001, ULE_I024, ULE_I037, ULE_I053, ULE_I057, ULE_I064, ULE_I068, ULE_I075, ULE_I080, ULE_I092 and LMG 10606 with *A. guillouiae* CIP 63.46^T^ (99.38–100%).

## Phylogenetic analysis

To determine the taxonomic position of the 11 isolates closely related to *A. guillouiae*, an additional phylogenetic analysis was performed using the *rpoB* gene (RNA polymerase β-subunit). The *rpoB* partial gene sequence was extracted from the ULE_I046^T^, ULE_I001, ULE_I024, ULE_I037, ULE_I053, ULE_I057, ULE_I064, ULE_I068, ULE_I075, ULE_I080 and ULE_I092 genomes and the *A. guillouiae* genomes from the National Center for Biotechnology Information (NCBI), by blastn alignment against the available *rpoB* sequences from the original description of *A. guillouiae* [8] (downloaded from nucleotide data associated to PMID 19661501). The partial *rpoB* sequences extracted and those from *A. guillouiae* isolates from Nemec *et al.* 2010 [8] were employed to build a phylogenetic tree by MAFFT (v.7) (https://mafft.cbrc.jp/alignment/server/index.html) [18] alignment and clustering using a Neighbour-Joining tree approach with 1,000 bootstrap values. Phylogenetic reconstruction based on *rpoB* revealed that three strains, RUH 2236, LUH 5606 and LUH 6980, previously assigned as *A. guillouiae* by Nemec *et al.* 2010 [8] clustered within our 11 isolates and were clearly separated from the *A. guillouiae* branch (Fig. S1). To further investigate this observation, strain LMG 10606 (=RUH 2236) was acquired from the BCCM/LMG Bacteria Collection and included in our analyses. DNA from this strain was extracted as indicated previously and sequenced by a GridION platform (Oxford Nanopore Technologies, Oxford, UK) using R10.4.1 flow cells and the Native Barcoding kit 24 (v.14). Adapters were removed from raw reads using Porechop (https://github.com/rrwick/Porechop) and assembly was done by Flye [19]. The quality of genome assembly was calculated by CheckM2 and Bakta Web [14] and taxonomic assignment was performed by GTDB-Tk (v.1.7.0) [16], being assigned as *Acinetobacter* species but not identified at the species level. The *16S rRNA* gene of this isolate was obtained as indicated previously. The obtained genome characteristics are in Table S1.

**Fig. 1.**
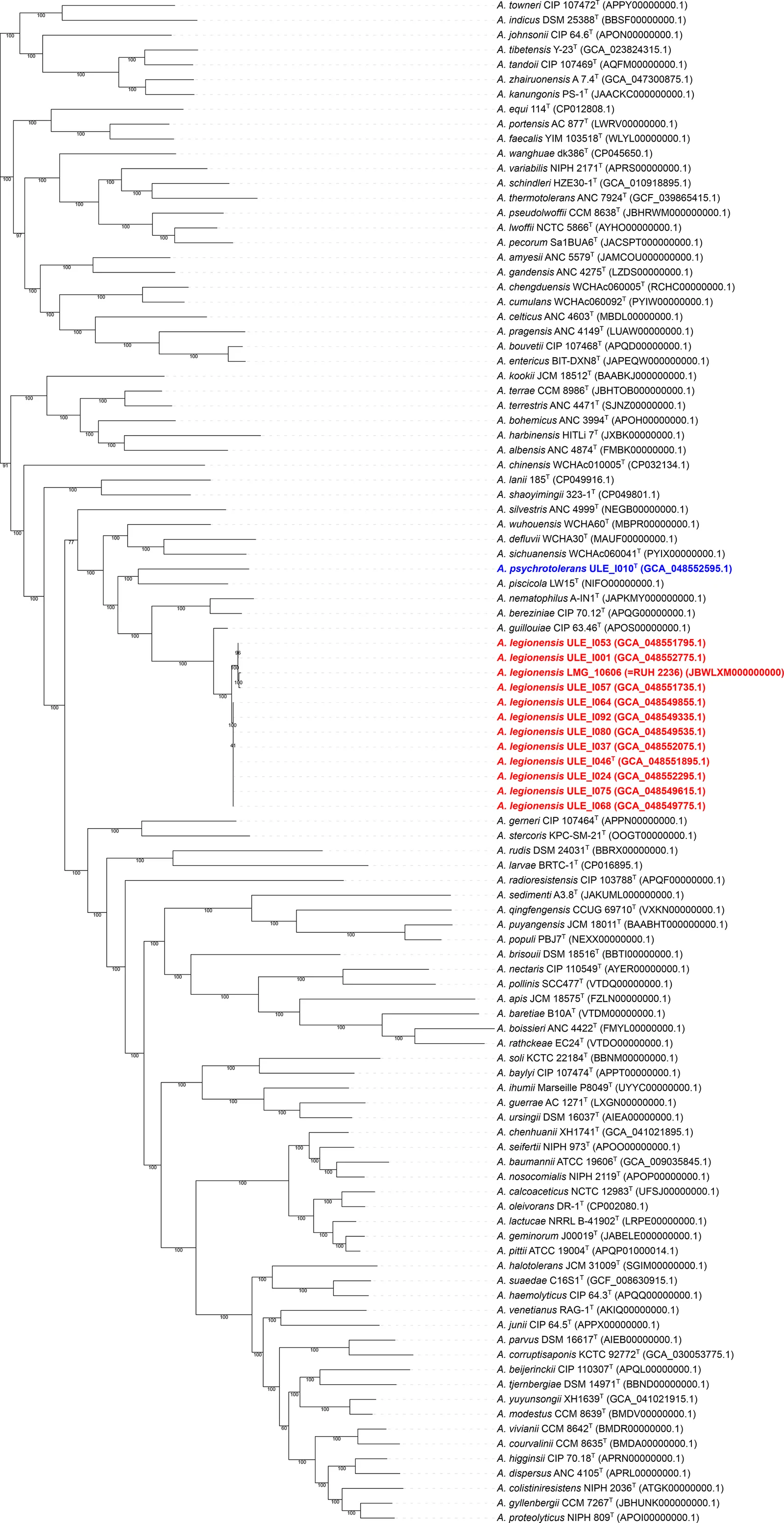
Phylogenetic tree based on the core genome of 12 isolates of *A. legionensis* sp. nov. (highlighted in red colour, 11 genomes from the present study and the strain LMG 10606), 1 strain of *A. psychrotolerans* sp. nov. (blue colour) and all the type strains of *Acinetobacter* species with validly published names.

As part of the genome comparison analysis, an additional phylogenomic tree based on an *Acinetobacter* genus core genome was constructed. To establish the placement of the 12 isolates under study and the strain LMG 10606, the reference genomes of 90 *Acinetobacter* species with names validly published under the ICNP (https://lpsn.dsmz.de/genus/acinetobacter, last accessed 23 March 2026) [2] were added to the analysis. Genomes of type strains of the 90 *Acinetobacter* species were downloaded from the NCBI (Table S3). Prokka (v.1.14.6) [20] and Panaroo (v.1.5.2) [21] were used to annotate the genomic sequences and compare the genes constituting the core genome, respectively. Then, FastTree (v.2.2.0) [22] was used for the alignment of the gene sequences and the construction of a maximum-likelihood core-genome phylogenetic tree. Branch support was estimated using SH-like local support values. The core-genome tree based on 86 genes revealed that the isolates ULE_I046^T^, ULE_I080, ULE_I037, ULE_I024, ULE_I075, ULE_I068, ULE_I092 and ULE_I064 comprised a cluster, including a subcluster comprising ULE_I057, ULE_I053, ULE_I001 and LMG 10606, separated from the type strains of known *Acinetobacter* species (Fig. 1), suggesting that the 11 isolates of this study and LMG 10606 belong to the same species within the genus *Acinetobacter*. These findings were consistent with the results obtained from *rpoB-*based phylogeny. Additionally, a maximum-likelihood core-genome phylogenetic tree, including these 11 isolates and LMG 10606, 48 *A*. *guillouiae* genomes obtained from the NCBI and taxonomically confirmed by GTDB-Tk, and the type strain of one closely related species, *Acinetobacter bereziniae* CIP 70.12^T^ was performed (Fig. S2). The 11 isolates and LMG 10606 clustered separately from *A. bereziniae* and all *A. guillouiae* genomes, suggesting that they are not members of the *A. guillouiae* species.

On the other hand, the strain ULE_I010^T^ stands in a separate branch from the rest of the genomes (Fig. 1), suggesting that it could also represent a novel *Acinetobacter* species.

In order to determine the clonality of *A. legionensis* sp. nov. isolates, a SNP-distance matrix was calculated by snp-dist (https://github.com/tseemann/snp-dists) using the *core_gene_alignment.aln* from Panaroo [21] outputs. There are clearly different clones detected by SNPs analysis for all the isolates except ULE_I037, ULE_I075 and ULE_I080 (with two or three SNPs between them) and ULE_I024 and ULE_I068 (no SNPs) (Table S4). The presence/absence of genes that do not constitute the core genome, obtained by Panaroo, showed that 24 and 5 genes were not equally distributed for both few-SNPs or no-SNPs clusters, ULE_I037-ULE_I075-ULE_I080 and ULE_I024-ULE_I068, respectively (Fig. S3). Other differences in gene content in the accessory genome for all *A. legionensis* sp. nov. isolates are presented in Fig. S4. This analysis confirms that the 12 isolates analysed (11 from ULE and the LMG 10606) do not belong to the same clone or strain.

## Overall genome relatedness indices

Overall genome relatedness indices (OGRIs) were evaluated for taxonomic determination of the new taxa following the proposed minimal standards for prokaryotic taxonomy [22]. In order to determine the classification of the isolates within the *Acinetobacter* genus, the average amino acid identity (AAI) values between the genomes of the 13 isolates (12 ULE and LMG 10606) and one reference genome per all *Acinetobacter* species with validly published and correct names (a total of 90) were calculated by EzAAI (v.1.2.3) [23]. All isolates showed AAI values equal to or higher than 68% (Fig. S5), above the proposed cut-off of 65–72% to define a bacterial genus [24, 25], confirming that the isolates belong to the genus *Acinetobacter*.

**Fig. 2.**
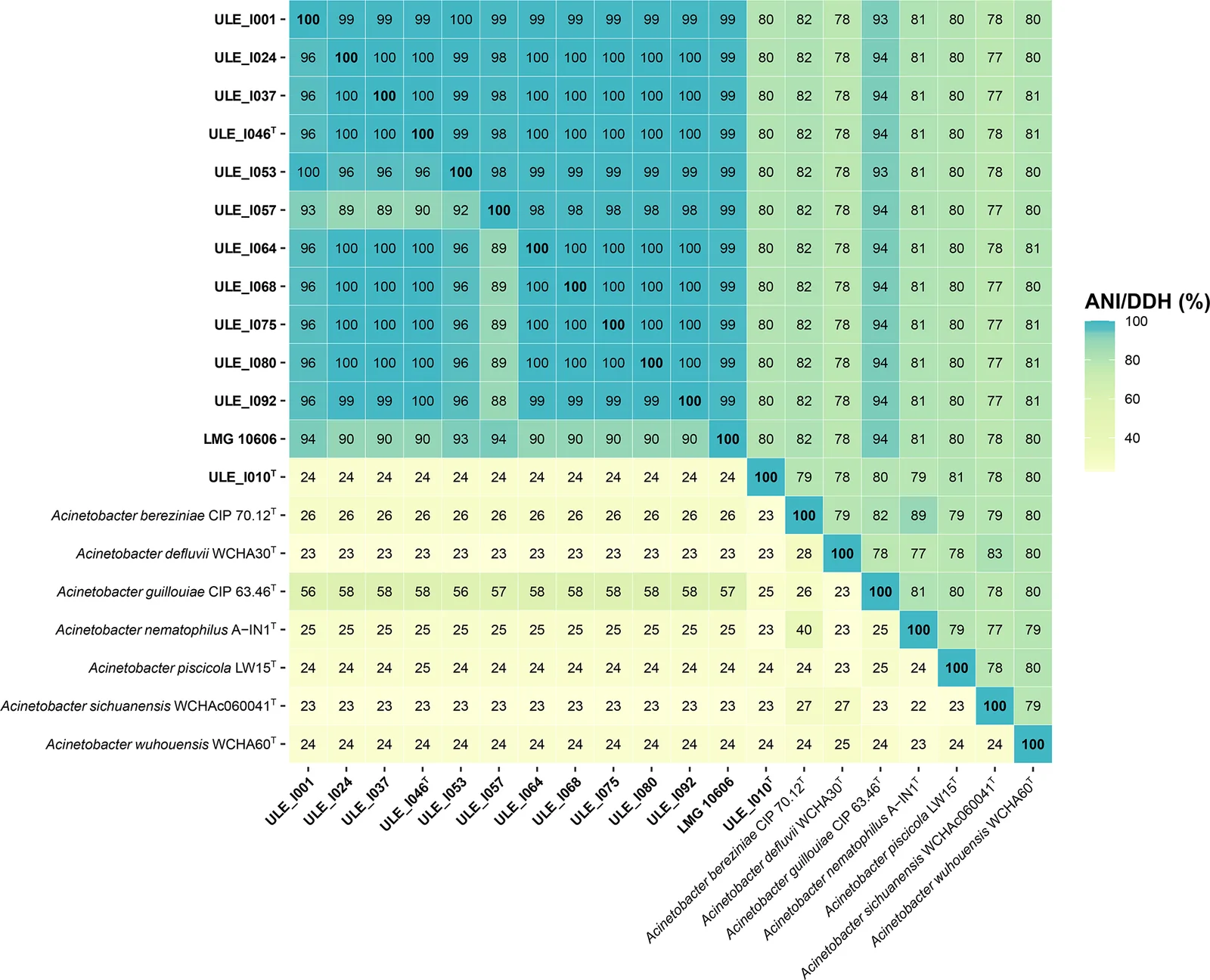
Percentage of ANI and dDDH for the isolates analysed in this study (including strain LMG 10606) and type strains of closely related *Acinetobacter* species. ANI values are shown on the top right of the diagonal and dDDH values on the bottom left.

Average nucleotide identities (ANIs) based on blast+ (ANIb) values were calculated using JSpeciesWS (https://jspecies.ribohost.com/jspeciesws/) [26], while the Genome-to-Genome Distance Calculator (GGDC v.3.0) (https://ggdc.dsmz.de/) [27], using the formula 2, was employed to determine the digital DNA–DNA hybridization (dDDH). ANIb and dDDH values were calculated using reference genomes of closely related *Acinetobacter* type strains according to the clustering obtained in the core-genome phylogenetic tree (Fig. 1): *A. guillouiae* CIP 63.46^T^, *A. bereziniae* CIP 70.12^T^, *Acinetobacter nematophilus* A-IN1^T^, *Acinetobacter wuhouensis* WCHA60^T^, *Acinetobacter piscicola* LW15^T^, *Acinetobacter sichuanensis* WCHAc060041^T^ and *Acinetobacter defluvii* WCHA30^T^. The isolates ULE_I046^T^, ULE_I001, ULE_I024, ULE_I037, ULE_I053, ULE_I057, ULE_I064, ULE_I068, ULE_I075, ULE_I080, ULE_I092 and LMG 10606 shared ANIb values higher than or equal to 98.30% (Fig. 2), which are above the suggested threshold of 95–96% proposed to distinguish between bacterial species [28–30]. This suggests that these 12 isolates belong to the same species, as it was already proposed after the observation of the core-genome and *rpoB* trees. The highest ANIb values of these 12 isolates with the closely related *Acinetobacter* type strains selected ranged from 93.39–93.95% (*A. guillouiae* CIP 63.46^T^), below the 95–96% threshold (Fig. 2). Likewise, dDDH values were higher than or equal to 88.40% between these 12 isolates, higher than the proposed threshold of 70% for prokaryotic species delineation [31, 32]. Conversely, the dDDH values between these isolates and the *Acinetobacter* type strains selected were below this threshold (22.8–57.9%) (Fig. 2). Therefore, ANI and dDDH values suggest that these 12 isolates belong to a single species distinct from all known *Acinetobacter* species.

**Fig. 3.**
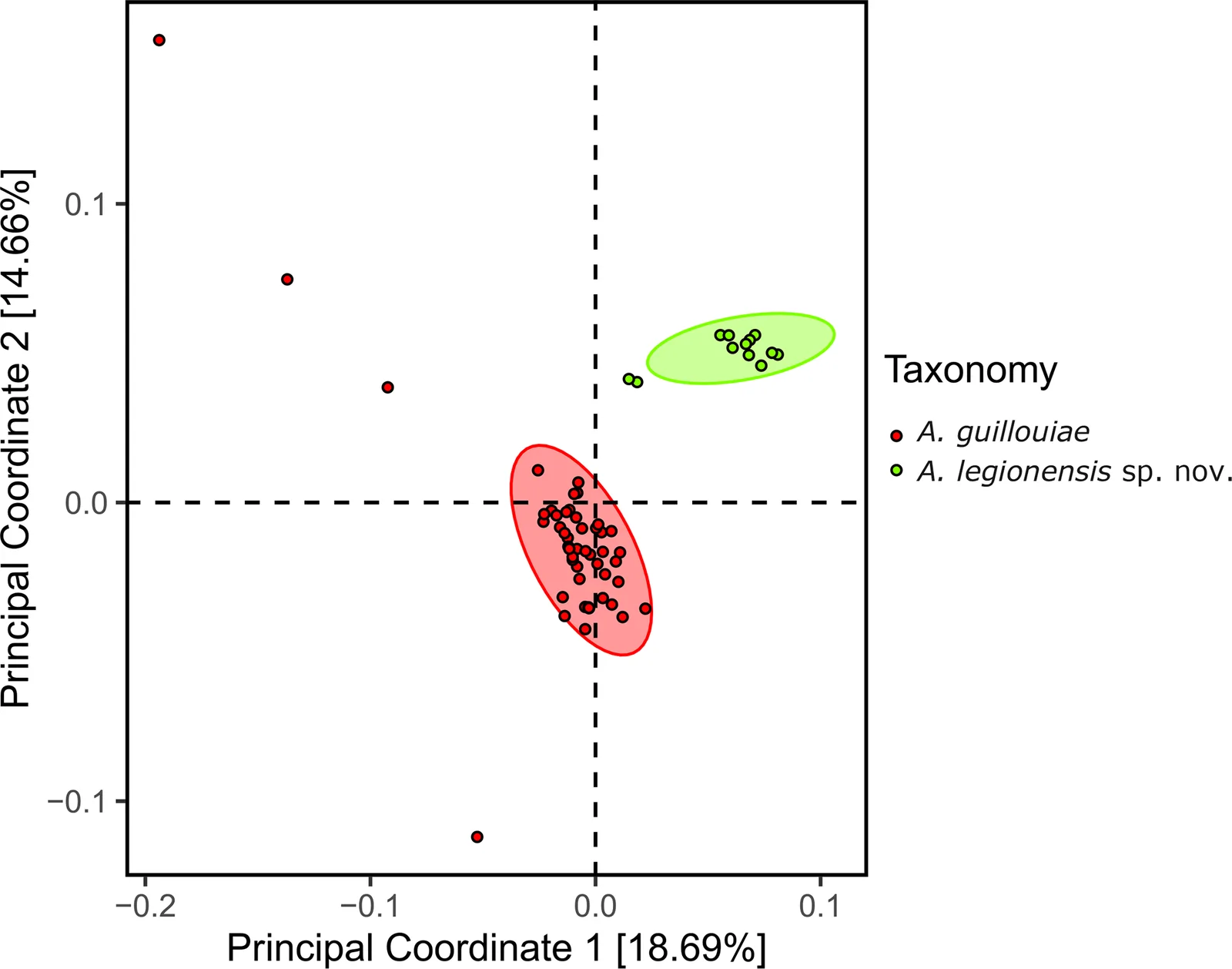
PCoA based on the abundance of metabolic functions between 48 genomes of *A. guillouiae* downloaded from NCBI and 12 genomes of the putative new species (*A. legionensis* sp. nov.; 11 genomes from the present study and the strain LMG 10606).

A principal coordinate analysis (PCoA) was performed based on the genomic functional annotations. The comparison at the functional level between these 12 isolates and all available genomes for the species *A. guillouiae* aimed to evaluate whether or not the isolates from this study exhibited a distinct functional profile relative to *A. guillouiae*. To this end, genomes were annotated using eggNOG-mapper (v.2) [33], and KEGG Orthology (KO) hits were extracted from the KO database (https://www.genome.jp/kegg/ko.html). An abundance matrix of each KO-code in each genome was generated and used to obtain a PCoA plot using the *ggplot* R package, and a clear separation between the genomes of the species *A. guillouiae* and the genomes of the 12 isolates evaluated in this study was observed (Fig. 3). This result shows that the two clusters have different functionalities, which supports the separation of the 12 isolates (11 from ULE and LMG 10606 strain) from *A. guillouiae*. Additionally, exclusive core genes specific for these isolates or for *A. guillouiae* were determined, taking a gene as a core gene for each of the two groups if it is present in at least 90% of the genomes from such group and in a maximum of 10% of the genomes of the other group. Following gene extraction and annotation, performed by Prokka (v.1.14.6) [20], a total of 62 and 167 genes were found as core genes exclusively for *A. guillouiae* and *A. legionensis* sp. nov. (11 ULE isolates and LMG 10606), respectively (Fig. S6, Table S5), with the core genes exclusive to *A. legionensis* sp. nov. being mainly related to the COG codes: [K] Transcription, [L] Replication, recombination and repair, [C] Energy production and conversion and [I] Lipid transport and metabolism, according to the results obtained by eggNOG-mapper (v.2) [33] (Table S4). Moreover, eight *mdc* genes (*mdcA*, *mdcB*, *mdcC*, *mdcD*, *mdcE*, *mdcG*, *mdcH*, *mdcR*) associated with malonate degradation were found within the core genes specifically present in *A. legionensis* sp. nov. and not in *A. guillouiae* (Table S5), which support, together with phenotypic assays exposed below, the possible value of malonate utilization as a diagnostic marker to differentiate *A. legionensis* sp. nov. and *A. guillouiae*.

Regarding the isolate ULE_I010^T^, ANIb (78.13–80.19%) and dDDH (22.80–24.60%) values between this genome and *Acinetobacter* type strains selected were below the 95–96% and 70% thresholds, respectively [28–32] (Fig. 2). Thus, these values suggest that the isolate ULE_I010^T^ belongs to a novel species within the genus *Acinetobacter*. Since ULE_I010^T^ was the unique isolate from the proposed new species obtained in our sampling campaign, a total of 17 metagenome-assembled genomes (MAGs) classified as *Acinetobacter* but unidentified at the species level downloaded from curated Food Metagenome Database (cFMD), the most comprehensive food metagenome database to date, that comprises >10,000 food MAGs from >2,500 food samples, including meat and meat preparations [34]; and other 10 *Acinetobacter* spp. MAGs recovered from meat processing environment metagenomes [35] were analysed in order to determine if they belong to the same branch as ULE_I010^T^, but no MAG belonging to this branch was found (data not shown). Overall, these findings justify a single-strain description for this novel *Acinetobacter* species.

## Phenotypic characterization

For further characterization of the newly proposed species, we tested the phenotypic features of 5 of the 11 isolates within *A. legionensis* sp. nov., including ULE_I046^T^, ULE_I024, ULE_I053, ULE_I057 and ULE_I075, and the single isolate ULE_I010^T^ constituting *A. psychrotolerans* sp. nov.

Growth capacity at different temperatures (5, 10, 15, 20, 25, 30, 35, 37 and 40 °C) was tested in Brain Heart Infusion broth (BHI, Merck KGaA) incubated for up to 7 days. NaCl tolerance was evaluated in Trypto-Casein Soy Broth (TSB, Condalab, Madrid, Spain) supplemented with 0, 0.5, 1.5, 3.0, 4.5 and 6.0% NaCl (wt/v), and incubated at 25 °C for 48 h. Growth at pH 4, 5, 6, 7, 8, 9 and 10 was tested in Luria–Bertani (LB) broth (VWR, Pennsylvania, USA), adjusted using HCl 2 N or NaOH 2 M to decrease or increase the pH, respectively, and incubated at 25 °C for 48 h. Growth was determined by absorbance measurements at 600 nm every 1 h using the SpectraMax^®^ iD3 (Molecular Devices, California, USA). Growth under anaerobic and microaerophilic conditions was evaluated in BHI agar (Merck KGaA) after 48 h of incubation at 25 °C, using Anaerocult^®^ A (Merck Millipore, Massachusetts, USA) and CampyGen^™^ 2.5 l packs (Thermo Scientific, Massachusetts, USA), respectively. Motility was evaluated in LB medium supplemented with 0.20, 0.25 and 0.30% (wt/v) agar according to Clemmer *et al*. [36]. Catalase activity was determined by mixing three to four colonies of a pure culture with a drop of H_2_O_2_ (10%, v/v), and oxidase activity was assessed using Oxidase Sticks (ITW Reagents Panreac, Barcelona, Spain). Gram staining was carried out using a Gram staining kit (bioMérieux, France), and cell morphology was visualized by light microscopy (Olympus CX41). Acid production from glucose and gelatin hydrolysis were evaluated using API 20 NE (bioMérieux). Haemolysis of sheep blood and citrate utilization (Simmons’) were tested according to Nemec *et al*. [37]. Tests for the assimilation of other carbon sources were performed using the basal mineral medium of Cruze *et al*. [38] supplemented with 0.1% (wt/v) carbon source, as previously described [37]. Other phenotypic analyses were carried out using the API 20 NE (bioMérieux) test and Biolog GEN III microplates (test for oxidation of carbon sources) (Protocol A; Biolog, Hayward, USA), according to the manufacturer’s instructions. The incubation temperature used in all these analyses was 25 °C. The results of phenotypic tests are given in the species descriptions. All results obtained by the Biolog GEN III system are shown in Table S6. Differential phenotypic characteristics of *A. legionensis* sp. nov. and *A. psychrotolerans* sp. nov. isolates and closely related species of the genus *Acinetobacter* based on the ANIb and dDDH analyses are shown in Table 2.

**Table 2.** Differential phenotypic characteristics of *A. legionensis* sp. nov. and *A. psychrotolerans* sp. nov. isolates and closely related species of the genus *Acinetobacter.* (1) *A. legionensis* sp. nov. (five isolates); (2) *A. psychrotolerans* sp. nov. (one isolate); (3) LMG 10606; (4) *A. guillouiae* (type strain LMG 988^T^); (5) *A. bereziniae*; (6) *A. piscicola*; (7) *A. wuhouensis*. Results of *A. legionensis* sp. nov., *A. psychrotolerans* sp. nov., LMG 10606 and the type strain of *A. guillouiae* were obtained in the present study. The results for *A. bereziniae*, *A. piscicola* and *A. wuhouensis* were obtained from https://szu.gov.cz/wp-content/anemec/Phenotype.pdf (version 8 January 2021).

Characteristic	1	2	3	4	5	6	7
Growth at:							
37 °C	+	–	+	+	+	–	–
35 °C	+	–	+	+	+	–	+
30 °C	+	–	+	+	+	+	+
Acidification of d-glucose	–	–	–	–	88(+)	–	–
Haemolysis of sheep blood	–	–	–	–	–	+	–
Assimilation of:							
Adipate	+	–	+	+	63(+)	–	–
Azelate	+	–	+	+	63(+)	–	–
2,3-Butanediol	+	–	+	+	+	–	+
Glutarate	+	–	+	+	+	–	+
Histamine	–	–	–	+	63(+)	–	–
Malonate	+	–	+	–	–	–	63(+)
Phenylacetate	–	–	–	+	25(–)	–	+
Capric acid	+	+	+	–	nd	nd	nd
Utilization of:							
4-Aminobutyrate	–	–	+	–	nd	nd	nd
l-Aspartate	80(+)	–	+	+	nd	nd	nd
l-Histidine	+	–	+	+	nd	nd	nd
d-Malate	20(+)	–	–	+	nd	nd	nd

+, All strains positive; –, all strains negative; nd, not determined. Numbers are percentages of strains with positive reaction, results for type strains are given in parentheses.

The five isolates of *A. legionensis* sp. nov. and the strain LMG 10606 were seen to have nearly identical phenotypic features (Table 2, Table S6). The combination of their ability to assimilate malonate and capric acid but not histamine and phenylacetate distinguished the six isolates (ULE and LMG 10606) from the type strain of *A. guillouiae* LMG 988^T^ (=CIP 63.46^T^). Most *A. guillouiae* strains are malonate-negative, as malonate utilization was observed in only 18% (3 out of 17) of the strains examined in the original description of *A. guillouiae* [8]. Notably, one of these three malonate-positive strains was LMG 10606, and the remaining two strains could correspond to LUH 5606 and LUH 6980, which based on the *rpoB* analysis, may also be reassigned to *A. legionensis* sp. nov. In addition, according to the Biolog test, all *A. legionensis* sp. nov. isolates, including LMG 10606, were unable to utilize γ-aminobutyric acid (GABA) (Table S6), whereas most *A. guillouiae* strains have the ability to degrade GABA [8], providing an additional phenotypic distinction. GABA metabolism has been associated with carbon and nitrogen metabolism, stress adaptation and potentially host-associated survival in *Acinetobacter* [39]. Therefore, this phenotypic difference may indicate distinct ecological adaptations between both species, although further research is required to determine its significance. The incapacity to produce acid from d-glucose, the absence of growth on histamine and the assimilation of malonate differentiated these isolates from *A. bereziniae* (Table 2). Strain ULE_I010^T^ differed from *A. guillouiae* LMG 988^T^ in eight characteristics (incapacity to grow over 30 °C, on adipate, azelate, 2,3-butanediol, glutarate, histamine and phenylacetate, and the ability to grow on capric acid), from *A. piscicola* in two characteristics (incapacity to grow over 30 °C and to lyse sheep blood), and from *A. wuhouensis* in five characteristics (incapacity to grow over 30 °C, on 2,3-butanediol, glutarate, malonate and phenylacetate) (Table 2). Additionally, the inability to utilize/oxidize l-aspartate, l-histidine and d-malate distinguished strain ULE_I010^T^ from *A. guillouiae* LMG 988^T^. In fact, the inability to grow over 30 °C by strain ULE_I010^T^ distinguishes it from the vast majority of known *Acinetobacter* species (https://szu.gov.cz/wp-content/anemec/Phenotype.pdf). MALDI-TOF MS was not able to reliably differentiate *A. legionensis* sp. nov. from *A. guillouiae* (Fig. S7)*,* however, considering the phenotypic results and those of the phylogenetic analyses based on the *rpoB* and core genome, as well as the calculation of genome relatedness by ANIb and dDDH, the isolates ULE_I046^T^, ULE_I001, ULE_I024, ULE_I037, ULE_I053, ULE_I057, ULE_I064, ULE_I068, ULE_I075, ULE_I080 and ULE_I092 are considered to represent a novel species within the genus *Acinetobacter,* for which the name *A. legionensis* sp. nov. is proposed.

Additionally, the *rpoB* and core-genome phylogenetic analyses, ANIb and dDDH values and phenotypic characteristics demonstrated that the isolate previously assigned to the species *A. guillouiae*, LMG 10606, belongs to the novel species *A. legionensis*. The *rpoB* analysis suggests that LUH 5606 and LUH 6980 could also be reassigned to *A. legionensis* sp. nov., but this fact has not been confirmed.

For ULE_I010^T^, unfortunately, only one isolate could be included in the current study resulting in lack of information about phenotypic variability within the novel species. However, the core-genome phylogenomic analysis, ANIb and dDDH values and phenotypic characteristics distinguished strain ULE_I010^T^ from other Acinetobacter species. Therefore, this strain represents another novel species within the genus *Acinetobacter*, for which the name *A. psychrotolerans* sp. nov. is proposed.

## Description of *Acinetobacter legionensis* sp. nov.

*Acinetobacter legionensis* (le.gio.nen’sis. N.L. masc. adj. *legionensis,* of or belonging to Legio, the Latin name for the city of *León*, Spain, where the isolates were collected).

Cells are Gram-stain-negative coccobacilli, non-motile, catalase-positive and oxidase-negative. Colonies are circular, light yellow and opaque and with 0.5–1.0 mm in diameter after 24 h of incubation at 25 °C on BHI agar plates, reaching 1–2 mm after 48 h of incubation under the same conditions. Growth occurs at 0–3% (wt/v) NaCl (one of the isolates up to 1.5%), pH 5–10 and 5–37 °C. Optimal growth was observed at 0% (wt/v) NaCl, pH 6–7 and 25 °C. All isolates can grow at microaerophilic and aerobic conditions and in BHI, TSB, LB and Mueller-Hinton media. Acid is not produced from d-glucose, gelatin is not hydrolysed and haemolysis is not observed on sheep blood agar. Acetate, adipate, azelate, 2,3-butanediol, ethanol, citrate (Simmons’), glutarate, malate, malonate and capric acid are utilized as sole sources of carbon with visible growth after 2 days of incubation. No growth occurs on l-arabinose, gentisate, d-glucose, histamine, l-leucine, l-ornithine, d-gluconate, phenylacetate, l-phenylalanine, d-ribose, d-mannose, d-mannitol, *N*-acetylglucosamine and maltose. Negative for nitrate reduction, indole production, arginine dihydrolase, urease, β-galactosidase and β-glucosidase hydrolysis. Isolates are able to utilize/oxidize (using the Biolog GEN III system) l-alanine, l-aspartate (80% of the isolates), l-glutamate, citrate, l-histidine, l-pyroglutamate, α-ketoglutarate, d-malate (20%), l-malate, bromo-succinate, propionate, acetate, methyl pyruvate (60%), l-lactate, α-hydroxybutyrate (40%), α-ketobutyrate and β-hydroxybutyrate.

Isolates belonging to this species were isolated from chicken, pork and beef fresh meat and meat preparation samples. The results of Puente *et al*. [7] suggest that this novel species of *Acinetobacter* is prevalent in meat products, as demonstrated by its presence in a minimum of 11 out 100 meat samples analysed. The type strain is ULE_I046^T^ (=CECT 31169^T^=CCP 535^T^), isolated from a mini chicken burger packaged under MAP obtained from a supermarket in *León*, Spain, in March 2022. Its genome has a size of 4.58 Mb and its G+C content is 37.7 mol%. The GenBank accession numbers for the *16S rRNA gene* and the whole-genome sequences are PV889005 and GCF_048551895.1, respectively.

## Description of *Acinetobacter psychrotolerans* sp. nov.

*Acinetobacter psychrotolerans* (psy.chro.to’le.rans. Gr. masc. adj. *psychros*, cold; L. pres. part. *tolerans*, tolerating; N.L. masc. part. adj. *psychrotolerans*, cold-tolerating).

Cells are Gram-stain-negative coccobacilli, non-motile, catalase-positive and oxidase-negative. Colonies are circular, light yellow and opaque and with <0.5 mm in diameter after 24 h at 25 °C on BHI agar plates, reaching 1–2 mm after 48 h incubation under the same conditions. Growth occurs at 0–0.5% NaCl (wt/v), pH 6–9 and 5–25 °C. Optimal growth was observed at 0% (wt/v) NaCl, pH 7 and 25 °C. This isolate can grow under microaerophilic and aerobic conditions and in BHI, TSB, LB and Mueller-Hinton media. Acid is not produced from d-glucose, gelatin is not hydrolysed and haemolysis is not observed on sheep blood agar. Acetate, ethanol, citrate (Simmons’) (weak positive result after 5 days of incubation), malate and capric acid are utilized as sole sources of carbon with visible growth after 2 days of incubation. No growth occurs on adipate, l-arabinose, azelate, 2,3-butanediol, gentisate, d-glucose, glutarate, histamine, l-leucine, malonate, l-ornithine, d-gluconate, phenylacetate, l-phenylalanine, d-ribose, d-mannose, d-mannitol, *N*-acetylglucosamine and maltose. Negative for nitrate reduction, indole production, arginine dihydrolase, urease, β-galactosidase and β-glucosidase hydrolysis. The strain is able to utilize/oxidize (using the Biolog GEN III system) l-alanine, l-glutamate, citrate, α-ketoglutarate, l-malate, bromo-succinate, propionate, acetate, methyl pyruvate, l-lactate, α-hydroxybutyrate, α-ketobutyrate, d-lactic acid methyl ester and β-hydroxybutyrate. The type strain is ULE_I010^T^ (=CECT 31168^T^=CCP 534^T^), isolated from a loin steak packaged under MAP obtained from a supermarket in *León*, Spain, in February 2022. Its genome has a size of 3.56 Mb and its G+C content is 37.2 mol%. The GenBank accession numbers for the *16S rRNA* gene and the whole-genome sequences are PV889002 and GCF_048552595.1, respectively.

## Supplementary material

10.1099/ijsem.0.007285 Fig. S1.

## Abbreviations

AAI: average amino acid identity
ANI: average nucleotide identity
ANIb: average nucleotide identities based on BLAST+
BHI: Brain Heart Infusion
BTS: bacterial test standard
dDDH: digital DNA–DNA hybridization
GABA: γ-aminobutyric acid
KO: KEGG Orthology
LB: Luria–Bertani
LPSN: list of prokaryotic names with standing in nomenclature
MAG: metagenome-assembled genome
MALDI-TOF MS: matrix-assisted laser desorption/ionization time-of-flight mass spectrometry
MAP: modified atmosphere packaging
MSP: main spectra profile
NCBI: National Center for Biotechnology Information
OGRIs: overall genome relatedness indices
PCoA: principal coordinate analysis
TSB: Trypto-Casein Soy Broth
