# Supplementary material for: Acinetobacter legionensis sp. nov. and Acinetobacter psychrotolerans sp. nov. isolated from different types of retail meat
Source: Int J Syst Evol Microbiol. 2026 Sep 22;76(9):007285. doi: 10.1099/ijsem.0.007285 (PMC13600464; doi:10.1099/ijsem.0.007285)
Supplement: Supplementary file 1 [file ijsem-76-07285-s001.pdf]

SUPPLEMENTARY MATERIAL

Acinetobacter legionensis sp. nov. and *Acinetobacter psychrotolerans* sp. nov. isolated from different types of retail meat

Alba Puente[a,b], Cristina Galisteo[c], José F. Cobo-Díaz[a,b], Filipa Grosso[d,e], Coral Barcenilla[a,b], Mercedes López[a,b], Miguel Prieto[a,b], Luisa Peixe[d,e], Avelino Álvarez-Ordóñez[a,b]

[a] Department of Food Science and Technology, Universidad de León, León, Spain

[b] Institute of Food Science and Technology (ICTAL), Universidad de León, León, Spain

[c] Department of Animal Health, Universidad de León, León, Spain

[d] UCIBIO–Applied Molecular Biosciences Unit, Faculty of Pharmacy, Department of Biological Sciences, Laboratory of Microbiology, University of Porto, Porto, Portugal

[e] Associate Laboratory i4HB, Institute for Health and Bioeconomy, Faculty of Pharmacy, University of Porto, Porto, Portugal

Corresponding author: José F. Cobo-Díaz. Department of Food Hygiene and Technology, Universidad de León, León, Spain. Phone: 0034 987291182; e-mail: jcobd@unileon.es

Table S1. Genome characteristics of the isolates constituting the novel species *A. legionensis* sp. nov. and *A. psychrotolerans* sp. nov.

Isolate	Genome accession nr.	*16S rRNA* gene accession nr.	Genome size (bp)	G+C content (mol%)	N50 (bp)	Contigs	Largest contig	CDS	tRNA	rRNA
A. legionensis ULE_I046^T	GCF_048551895.1	PV889005	4582574	37.7	122954	160	351651	4238	74	9
A. legionensis ULE_I001	GCF_048552775.1	PV889001	4585795	37.7	158256	146	476223	4271	70	4
A. legionensis ULE_I024	GCF_048552295.1	PV889003	4526332	37.7	134031	131	346389	4230	74	6
A. legionensis ULE_I037	GCF_048552075.1	PV889004	4517785	37.7	160665	136	274839	4229	74	9
A. legionensis ULE_I053	GCF_048551795.1	PV889006	4698705	37.7	209238	355	476293	4343	72	4
A. legionensis ULE_I057	GCF_048551735.1	PV889007	4406595	37.7	186866	128	408097	4154	71	3
A. legionensis ULE_I064	GCF_048549855.1	PV889008	4595206	37.6	93544	201	293579	4247	71	9
A. legionensis ULE_I068	GCF_048549775.1	PV889009	4532026	37.7	131506	154	346391	4236	74	9
A. legionensis ULE_I075	GCF_048549615.1	PV889010	4519175	37.7	146523	141	325937	4237	74	7
A. legionensis ULE_I080	GCF_048549535.1	PV889011	4564985	37.7	160672	286	325945	4252	74	10
A. legionensis ULE_I092	GCF_048549335.1	PV889012	4702609	37.7	195159	236	343907	4449	76	9
A. legionensis LMG 10606	JBWLXM000000000	PZ251116	4392684	37.9	4389143	2	4389143	3952	82	21
A. psychrotolerans ULE_I010^T	GCF_048552595.1	PV889002	3562775	37.2	170513	90	365995	3278	73	3

Table S2. *16S rRNA* gene sequences from representative *Acinetobacter* species employed in blastn database to extract *16S rRNA* sequences for new sequenced genomes.

Nucleotide ID	Species	Strain
NR_145641.1	*Acinetobacter albensis*	ANC 4874
NR_115739.1	*Acinetobacter antiviralis*	KNF2022
NR_133952.1	*Acinetobacter apis*	HYN18
NR_180956.1	*Acinetobacter baretiae*	B10A
NR_117620.1	*Acinetobacter baumannii*	ATCC 19606
NR_116845.1	*Acinetobacter baumannii*	CIP 70.34
NR_117677.1	*Acinetobacter baumannii*	DSM 30007
NR_026206.1	*Acinetobacter baumannii*	DSM 30007
NR_113237.1	*Acinetobacter baumannii*	JCM 6841
NR_119358.1	*Acinetobacter baumannii*	ATCC 19606
NR_115042.2	*Acinetobacter baylyi*	B2
NR_028848.1	*Acinetobacter baylyi*	B2
NR_042234.1	*Acinetobacter beijerinckii*	58a
NR_117625.1	*Acinetobacter bereziniae*	ATCC 17924
NR_178628.1	*Acinetobacter bohemicus*	ANC 3994
NR_118409.1	*Acinetobacter boissieri*	SAP 284.1
NR_028851.1	*Acinetobacter bouvetii*	4B02
NR_117628.1	*Acinetobacter bouvetii*	DSM 14964
NR_115871.1	*Acinetobacter brisouii*	5YN5-8
NR_117619.1	*Acinetobacter calcoaceticus*	ATCC 23055
NR_042387.1	*Acinetobacter calcoaceticus*	NCCB 22016
NR_114958.1	*Acinetobacter calcoaceticus*	ATCC 23055
NR_114922.1	*Acinetobacter calcoaceticus*	CIP 81.8
NR_114921.1	*Acinetobacter calcoaceticus*	LMG 1046
NR_113343.1	*Acinetobacter calcoaceticus*	JCM 6842
NR_119113.1	*Acinetobacter calcoaceticus*	DSM 30006 = CIP 81.8
NR_119357.1	*Acinetobacter calcoaceticus*	ATCC 23055
NR_174302.1	*Acinetobacter chengduensis*	WCHAc060005
NR_165666.1	*Acinetobacter chinensis*	WCHAc010005
NR_148843.1	*Acinetobacter courvalinii*	ANC 3623
NR_156989.1	*Acinetobacter defluvii*	WCHA30
NR_148844.1	*Acinetobacter dispersus*	ANC 4105
NR_148643.1	*Acinetobacter equi*	114
NR_133953.1	*Acinetobacter gandensis*	UG 60467
NR_181169.1	*Acinetobacter geminorum*	J00019
NR_028852.1	*Acinetobacter gerneri*	9A01 = DSM 14967 = CIP 107464 = MTCC 9824
NR_117627.1	*Acinetobacter gerneri*	DSM 14967 = CIP 107464 = MTCC 9824
NR_179028.1	*Acinetobacter guerrae*	AC 1271
NR_117626.1	*Acinetobacter guillouiae*	ATCC 11171
NR_042026.1	*Acinetobacter gyllenbergii*	RUH 422
NR_117622.1	*Acinetobacter haemolyticus*	ATCC 17906
NR_026207.1	*Acinetobacter haemolyticus*	DSM 6962
NR_119359.1	*Acinetobacter haemolyticus*	ATCC 17906
NR_149800.1	*Acinetobacter halotolerans*	R160
NR_178590.1	*Acinetobacter harbinensis*	HITLi 7
NR_179446.1	*Acinetobacter ihumii*	Marseille-P8049
NR_133954.1	*Acinetobacter indicus*	1NM-4
NR_117784.1	*Acinetobacter indicus*	A648 = CIP 110367
NR_117624.1	*Acinetobacter johnsonii*	ATCC 17909
NR_164627.1	*Acinetobacter johnsonii*	ATCC 17909

NR_119114.1	*Acinetobacter johnsonii*	DSM 6963
NR_044975.1	*Acinetobacter johnsonii*	Mannheim 3865/60
NR_117623.1	*Acinetobacter junii*	ATCC 17908
NR_026208.1	*Acinetobacter junii*	DSM 6964
NR_119360.1	*Acinetobacter junii*	Mannheim 2723/59
NR_181459.1	*Acinetobacter kanungonis*	PS-1
NR_135727.1	*Acinetobacter kookii*	11-0202
NR_116714.1	*Acinetobacter kyonggiensis*	KSL5401-037
NR_152004.1	*Acinetobacter lactucae*	NRRL B-41902
NR_152082.1	*Acinetobacter lactucae*	JVAP01
NR_180591.1	*Acinetobacter lanii*	185
NR_156845.1	*Acinetobacter larvae*	BRTC-1
NR_026209.1	*Acinetobacter lwoffii*	DSM 2403
NR_113346.1	*Acinetobacter lwoffii*	JCM 6840
NR_115298.1	*Acinetobacter marinus*	SW-3
NR_148845.1	*Acinetobacter modestus*	NIPH 236
NR_145841.1	*Acinetobacter movanagherensis*	Movanagher 4
NR_118408.1	*Acinetobacter nectaris*	CIP 110549 = SAP 763.2
NR_117931.1	*Acinetobacter nosocomialis*	RUH 2376
NR_102814.1	*Acinetobacter oleivorans*	DR1
NR_116039.1	*Acinetobacter oleivorans*	DR1
NR_180005.1	*Acinetobacter oryzae*	B23
NR_178416.1	*Acinetobacter oryzae*	B23
NR_025425.1	*Acinetobacter parvus*	LUH4616
NR_159919.1	*Acinetobacter piscicola*	LW15
NR_116774.1	*Acinetobacter pittii*	DSM 21653 = CIP 70.29
NR_117621.1	*Acinetobacter pittii*	DSM 21653 = ATCC 19004
NR_117930.1	*Acinetobacter pittii*	LMG 1035
NR_178762.1	*Acinetobacter plantarum*	THG-SQM11
NR_145864.1	*Acinetobacter populi*	PBJ7
NR_179027.1	*Acinetobacter portensis*	AC 877
NR_152069.1	*Acinetobacter pragensis*	ANC 4149
NR_148846.1	*Acinetobacter proteolyticus*	NIPH 809
NR_181286.1	*Acinetobacter pseudolwoffii*	CCM 8638
NR_169498.1	*Acinetobacter pullicarnis*	S23
NR_109507.1	*Acinetobacter puyangensis*	BQ4-1
NR_133685.1	*Acinetobacter qingfengensis*	2BJ1
NR_114074.1	*Acinetobacter radioresistens*	NBRC 102413
NR_026210.1	*Acinetobacter radioresistens*	FO-1
NR_180953.1	*Acinetobacter rathckeae*	EC24
NR_178633.1	*Acinetobacter refrigeratoris*	WB1
NR_180534.1	*Acinetobacter rongchengensis*	WCHAc060115
NR_115988.1	*Acinetobacter rudis*	G30
NR_025412.1	*Acinetobacter schindleri*	LUH5832
NR_134684.1	*Acinetobacter seifertii*	LUH 1472
NR_115299.1	*Acinetobacter seohaensis*	SW-100
NR_116069.1	*Acinetobacter septicus*	AK001
NR_116070.1	*Acinetobacter septicus*	AK001
NR_116071.1	*Acinetobacter septicus*	AK001
NR_181115.1	*Acinetobacter shaoyimingii*	323-1
NR_180592.1	*Acinetobacter shaoyimingii*	323-1
NR_171416.1	*Acinetobacter sichuanensis*	WCHAc060041
NR_181930.1	*Acinetobacter silvestris*	ANC 4999
NR_044454.1	*Acinetobacter soli*	B1

NR_181117.1	*Acinetobacter stercoris*	KPC-SM-21
NR_028853.1	*Acinetobacter tandoii*	4N13
NR_117630.1	*Acinetobacter tandoii*	DSM 14970
NR_117508.1	*Acinetobacter tandoii*	DSM 14970
NR_180533.1	*Acinetobacter tianfuensis*	WCHAc060012
NR_117629.1	*Acinetobacter tjernbergiae*	DSM 14971
NR_117193.1	*Acinetobacter tjernbergiae*	DSM 14971
NR_028850.1	*Acinetobacter tjernbergiae*	7N16
NR_028849.1	*Acinetobacter towneri*	AB1110
NR_025392.1	*Acinetobacter ursingii*	LUH3792
NR_134685.1	*Acinetobacter variabilis*	NIPH 2171
NR_042049.1	*Acinetobacter venetianus*	RAG-1 = CIP 110063 = ATCC 31012
NR_148847.1	*Acinetobacter vivianii*	NIPH 2168
NR_180336.1	*Acinetobacter wanghuae*	dk386
NR_179145.1	*Acinetobacter wuhouensis*	WCHA60

Table S3. Reference genomes of 90 *Acinetobacter* species with validly published correct name and their corresponding accession numbers from NCBI database employed to phylogenomic analyses.

Type strains	Accession numbers NCBI
Acinetobacter albensis ANC 4874^{T}	FMBK00000000.1
Acinetobacter amyesii ANC 5579^{T}	JAMCOU000000000.1
Acinetobacter apis JCM 18575^{T}	GCA_054782275.1
Acinetobacter baretiae B10A^{T}	VTDM00000000.1
Acinetobacter baumannii ATCC 19606^{T}	GCA_009035845.1
Acinetobacter baylyi CIP 107474^{T}	APPT00000000.1
Acinetobacter beijerinckii CIP 110307^{T}	APQL00000000.1
Acinetobacter bereziniae CIP 70.12^{T}	APQG00000000.1
Acinetobacter bohemicus ANC 3994^{T}	APOH00000000.1
Acinetobacter boissieri ANC 4422^{T}	FMYL00000000.1
Acinetobacter bouvetii CIP 107468^{T}	APQD00000000.1
Acinetobacter brisouii DSM 18516^{T}	BBTI00000000.1
Acinetobacter calcoaceticus NCTC 12983^{T}	UFSJ00000000.1
Acinetobacter celticus ANC 4603^{T}	MBDL00000000.1
Acinetobacter chengduensis WCHAc060005^{T}	RCHC00000000.1
Acinetobacter chenhuanii XH1741^{T}	GCA_041021895.1
Acinetobacter chinensis WCHAc010005^{T}	CP032134.1
Acinetobacter colistiniresistens NIPH 2036^{T}	ATGK00000000.1
Acinetobacter corruptisaponis KCTC 92772^{T}	GCA_030053775.1
Acinetobacter courvalinii CCM 8635^{T}	BMDA00000000.1
Acinetobacter cumulans WCHAc060092^{T}	PYIW00000000.1
Acinetobacter defluvii WCHA30^{T}	MAUF00000000.1
Acinetobacter dispersus ANC 4105^{T}	APRL00000000.1
Acinetobacter entericus BIT DXN8^{T}	JAPEQW000000000.1
Acinetobacter equi 114^{T}	CP012808.1
Acinetobacter faecalis YIM 103518^{T}	WLYL00000000.1
Acinetobacter gandensis ANC 4275^{T}	LZDS00000000.1
Acinetobacter geminorum J00019^{T}	JABELE000000000.1
Acinetobacter gerneri CIP 107464^{T}	APPN00000000.1
Acinetobacter guerrae AC 1271^{T}	LXGN00000000.1
Acinetobacter guillouiae CIP 63.46^{T}	APOS00000000.1
Acinetobacter gyllenbergii CCM 7267^{T}	JBHUNK000000000.1
Acinetobacter haemolyticus CIP 64.3^{T}	APQQ00000000.1
Acinetobacter halotolerans JCM 31009^{T}	SGIM00000000.1
Acinetobacter harbinensis HITLi 7^{T}	JXBK00000000.1
Acinetobacter higginsii CIP 70.18^{T}	APRN00000000.1
Acinetobacter ihumii Marseille P8049^{T}	UYYC00000000.1
Acinetobacter indicus DSM 25388^{T}	BBSF00000000.1
Acinetobacter johnsonii CIP 64.6^{T}	APON00000000.1
Acinetobacter junii CIP 64.5^{T}	APPX00000000.1
Acinetobacter kanungonis PS 1^{T}	JAACKC000000000.1
Acinetobacter kookii JCM 18512^{T}	BAABKJ000000000.1
Acinetobacter lactucae NRRL B-41902^{T}	LRPE00000000.1
Acinetobacter lanii 185^{T}	CP049916.1
Acinetobacter larvae BRTC-1^{T}	CP016895.1
Acinetobacter lwoffii NCTC 5866^{T}	AYHO00000000.1
Acinetobacter modestus CCM 8639^{T}	BMDV00000000.1
Acinetobacter nectaris CIP 110549	AYER00000000.1

Acinetobacter nematophilus A-IN1^T	JAPKMY000000000.1
Acinetobacter nosocomialis NIPH 2119^T	APOP00000000.1
Acinetobacter oleivorans DR-1^T	CP002080.1
Acinetobacter parvus DSM 16617^T	AIEB00000000.1
Acinetobacter pecorum Sa1BUA6^T	JACSPT000000000.1
Acinetobacter piscicola LW15^T	NIFO00000000.1
Acinetobacter pittii ATCC 19004^T	APQP01000014.1
Acinetobacter pollinis SCC477^T	VTDQ00000000.1
Acinetobacter populi PBJ7^T	NEXX00000000.1
Acinetobacter portensis AC 877^T	LWRV00000000.1
Acinetobacter pragensis ANC 4149^T	LUAW00000000.1
Acinetobacter proteolyticus NIPH 809^T	APOI00000000.1
Acinetobacter pseudolwoffii CCM 8638^T	JBHRWM000000000.1
Acinetobacter puyangensis JCM 18011^T	BAABHT000000000.1
Acinetobacter qingfengensis CCUG 69710^T	VXKN00000000.1
Acinetobacter radioresistens CIP 103788^T	APQF00000000.1
Acinetobacter rathckeae EC24^T	VTDO00000000.1
Acinetobacter rudis DSM 24031^T	BBRX00000000.1
Acinetobacter schindleri HZE30-1^T	GCA_010918895.1
Acinetobacter sedimenti A3.8^T	JAKUML000000000.1
Acinetobacter seifertii NIPH 973^T	APOO00000000.1
Acinetobacter shaoyimingii 323-1^T	CP049801.1
Acinetobacter sichuanensis WCHAc060041^T	PYIX00000000.1
Acinetobacter silvestris ANC 4999^T	NEGB00000000.1
Acinetobacter soli KCTC 22184^T	BBNM00000000.1
Acinetobacter stercoris KPC-SM-21^T	OOGT00000000.1
Acinetobacter suaedae C16S1^T	GCF_008630915.1
Acinetobacter tandoii CIP 107469^T	AQFM00000000.1
Acinetobacter terrae CCM 8986^T	JBHTOB000000000.1
Acinetobacter terrestris ANC 4471^T	SJNZ00000000.1
Acinetobacter thermotolerans ANC 7924^T	GCF_039865415.1
Acinetobacter tibetensis Y-23^T	GCA_023824315.1
Acinetobacter tjernbergiae DSM 14971^T	BBND00000000.1
Acinetobacter towneri CIP_107472^T	APPY00000000.1
Acinetobacter ursingii DSM 16037^T	AIEA00000000.1
Acinetobacter variabilis NIPH 2171^T	APRS00000000.1
Acinetobacter venetianus RAG-1^T	AKIQ00000000.1
Acinetobacter vivianii CCM 8642^T	BMDR00000000.1
Acinetobacter wanghuae dk386^T	CP045650.1
Acinetobacter wuhouensis WCHA60^T	MBPR00000000.1
Acinetobacter yuyunsongii XH1639^T	GCA_041021915.1
Acinetobacter zhairuonensis A 7.4^T	GCA_047300875.1

Table S4. Number of SNPs detected between *A. legionensis* sp. nov. genomes.

snp-dists 0.8.2	ULE_I001	ULE_I024	ULE_I037	ULE_I046^T	ULE_I053	ULE_I057	ULE_I064	ULE_I068	ULE_I075	ULE_I080	ULE_I092	LMG 10606
**ULE_I001**	0	14337	14337	14271	58	18852	14292	14337	14323	14327	14197	17971
**ULE_I024**	14337	0	141	321	14317	30889	480	0	134	128	298	30573
**ULE_I037**	14337	141	0	310	14311	30404	469	125	3	2	287	30365
**ULE_I046**	14271	321	310	0	14286	30368	453	321	310	310	271	30166
**ULE_I053**	58	14317	14311	14286	0	19284	14268	14312	14298	14302	14172	18246
**ULE_I057**	18852	30889	30404	30368	19284	0	30720	30350	30784	30369	30245	18865
**ULE_I064**	14292	480	469	453	14268	30720	0	480	469	469	332	30382
**ULE_I068**	14337	0	125	321	14312	30350	480	0	125	125	298	30173
**ULE_I075**	14323	134	3	310	14298	30784	469	125	0	2	287	30530
**ULE_I080**	14327	128	2	310	14302	30369	469	125	2	0	288	30184
**ULE_I092**	14197	298	287	271	14172	30245	332	298	287	288	0	30090
**LMG 10606**	17971	30573	30365	30166	18246	18865	30382	30173	30530	30184	30090	0

Table S5. Core genes found for *A. guillouiae* and *A. legionensis* sp. nov. genomes. Prokka gene-name, COG_category and preferred name (by eggnog-mapper) are indicated.

Prokka core genes *A. guillouiae*	COG_category	Preferred_name
alkJ_1~~~betA_2~~~alkJ_2~~~alkJ_4~~~betA_1~~~alkJ_3~~~betA	E	*alkJ*
dap	V	*ampH*
argE~~~argE_1	E	*argE*
cysJ~~~cysJ_1	P	*cysJ*
fdhD	C	*fdhD*
group_4498	S	*fimA*
gstB_1~~~gstA~~~gstB_2~~~gstB_3	O	*gst*
moaA_1~~~moaA_2~~~moaA2	H	*moaA*
moaC_2	H	*moaC*
moaE1	H	*moaE*
mobA_2~~~mobA_1~~~mobA	H	*mobA*
group_3810	P	*modB*
group_4990	H	*moeA*
puuC~~~puuC_2~~~puuC_1	C	*puuC2*
ydeP	C	*ydeP*
cysB~~~gltC_1	K	*yvbU*
group_3755	-	-
group_4981	S	-
group_3183	S	-
group_3175	EGP	-
group_3184	P	-
group_5891	-	-
nicR	K	-
group_430	O	-
group_5208	-	-
group_5369	M	-
group_5112	S	-
group_4328	H	-
group_5698	S	-
nicX	E	-
group_3304	S	-
nfdA_1~~~nfdA~~~nfdA_3	S	-
group_3319	-	-
group_3181	-	-
group_4354	S	-
paaG_2~~~paaG_1~~~paaG_3	I	-
pgrR~~~pgrR_4~~~pgrR_3~~~pgrR_1~~~pgrR_5	K	-
czcO_1~~~czcO~~~czcO_2	P	-
cnbA_1~~~cnbA~~~cnbA_2	C	-
group_4922	U	-
group_3558	-	-
group_3742	J	-
mprA~~~mprA_1~~~slyA~~~mprA_2	K	-
yjiA	S	-
group_4889	-	-
group_4352	-	-

yobA~~~yobA_1~~~yobA_2	S	-
group_4895	H	-
group_5607	P	-
group_3775	-	-
group_3046	-	-
group_5343	-	-
yhaI	S	-
group_5859	-	-
group_3957	-	-
group_5160	S	-
group_5241	O	-

**Prokka core genes *A. legionensis* sp. nov.**	**COG_category**	**Preferred_name**
iolA	EG	*alh-8*
group_1378	I	*cpo*
cpo_2	I	*cpo*
group_6544	S	*exoR*
group_3231	IQ	*IV02_09705*
aptA	G	*IV02_09715*
aptB	G	*IV02_09720*
group_1785	S	*lys*
group_7345	S	*madL*
group_3084	S	*madM*
madA	I	*mdcA*
citG	H	*mdcB*
mdcC	C	*mdcC*
madC	I	*mdcD*
madD	I	*mdcE*
mdcG	H	*mdcG*
fabD_2	I	*mdcH*
cynR_2	K	*mdcR*
modA	P	*modA*
modB	P	*modB*
modC	P	*modC*
moeA	H	*moeA*
otsA	G	*otsA*
otsB	G	*otsB*
perR	K	*perR*
ppsC_1	C	*qor2*
rutE~~~rutE_3~~~rutE_2	C	*rutE*
tdhA	P	*tdhA*
umuD_1	KT	*umuD*
ybjJ_1	G	*ybjJ_2*
group_989	-	-
group_2962	NU	-
group_3151	L	-
group_2565	-	-

group_7096	L	-
hxlR	K	-
group_647	S	-
rhaS_3	K	-
group_1164	S	-
group_3934	M	-
oprB_4	M	-
group_2608	S	-
group_427	S	-
group_5065	-	-
group_3350	S	-
group_552	-	-
group_717	S	-
group_7665	-	-
group_7898	S	-
group_7588	S	-
hchA_2	S	-
group_2552	-	-
group_5917	K	-
group_6453	-	-
group_3735	S	-
group_774	-	-
namA_3	C	-
group_5533	-	-
group_7242	-	-
group_2928	-	-
group_25	L	-
group_1036	-	-
group_7472	-	-
group_1958	S	-
group_2721	I	-
group_2058	-	-
jefA	EGP	-
group_2394	-	-
group_7610	-	-
group_6349	L	-
group_4148	M	-
group_7215	S	-
group_2365	-	-
COQ3~~~COQ3_1	J	-
group_2468	EG	-
calB_1	C	-
rhaS_2	K	-
pigA_2	P	-
group_2723	K	-
group_7914	M	-
group_7068	S	-
group_4944	NU	-
group_7259	-	-

group_6829	M	-
group_3005	S	-
group_7139	S	-
nicT	G	-
group_2523	-	-
group_108	-	-
group_4321	S	-
group_6257	S	-
group_5030	NU	-
group_4778	S	-
group_5348	S	-
group_7069	S	-
fecR_2	PT	-
group_7114	-	-
group_2675	-	-
group_2649	S	-
group_7230	S	-
echA8_1	I	-
group_7839	L	-
ycjY_1	S	-
group_2560	-	-
namA_2	C	-
group_150	I	-
group_6221	M	-
hmrR_1	K	-
group_7308	L	-
group_4046	-	-
group_672	K	-
group_7492	S	-
group_7270	-	-
group_2374	K	-
group_867	-	-
group_349	L	-
group_6765	U	-
group_428	S	-
ycjY_2	S	-
kstR2_2	K	-
group_7609	-	-
group_7055	-	-
mmsB_2	I	-
group_2154	I	-
group_223	-	-
group_3425	S	-
group_2579	S	-
group_6258	S	-
group_2161	C	-

Table S6. Results of the isolates analysed in the study using the Biolog GEN III system. +, positive oxidative substrate utilization; -, negative oxidative substrate utilization.

Species	*A. psychrotolerans*	*A. legionensis*	*A. legionensis*	*A. legionensis*	*A. legionensis*	*A. legionensis*	*A. legionensis*	*A. guillouiae*
Strain	ULE_I010^T	ULE_I046^T	ULE_I02 4	ULE_I053	ULE_I057	ULE_075	LMG 10606	LMG 988^T
Dextrin	-	-	-	-	-	-	-	-
D-maltose	-	-	-	-	-	-	-	-
D-trehalose	-	-	-	-	-	-	-	-
D-cellobiose	-	-	-	-	-	-	-	-
Gentiobiose	-	-	-	-	-	-	-	-
Sucrose	-	-	-	-	-	-	-	-
D-turanose	-	-	-	-	-	-	-	-
Stachyose	-	-	-	-	-	-	-	-
D-raffinose	-	-	-	-	-	-	-	-
α-D-lactose	-	-	-	-	-	-	-	-
D-melibiose	-	-	-	-	-	-	-	-
β-methyl-D-glucoside	-	-	-	-	-	-	-	-
D-salicin	-	-	-	-	-	-	-	-
N-acetyl-D-glucosamine	-	-	-	-	-	-	-	-
N-acetyl-β-D-mannosamine	-	-	-	-	-	-	-	-
N-acetyl-D-galactosamine	-	-	-	-	-	-	-	-
N-acetyl neuraminic acid	-	-	-	-	-	-	-	-
α-D-glucose	-	-	-	-	-	-	-	-
D-mannose	-	-	-	-	-	-	-	-
D-fructose	-	-	-	-	-	-	-	-
D-galactose	-	-	-	-	-	-	-	-
3-methyl glucose	-	-	-	-	-	-	-	-
D-fucose	-	-	-	-	-	-	-	-
L-fucose	-	-	-	-	-	-	-	-
L-rhamnose	-	-	-	-	-	-	-	-
Inosine	-	-	-	-	-	-	-	-
D-sorbitol	-	-	-	-	-	-	-	-
D-mannitol	-	-	-	-	-	-	-	-
D-arabitol	-	-	-	-	-	-	-	-

Myo-inositol	-	-	-	-	-	-	-	-
Glycerol	-	-	-	-	-	-	-	-
D-glucose-6-PO4	-	-	-	-	-	-	-	-
D-fructose-6-PO4	-	-	-	-	-	-	-	-
D-aspartic acid	-	-	-	-	-	-	-	-
D-serine	+	+	+	+	+	+	+	+
Gelatin	-	-	-	-	-	-	-	-
Glycyl-L-proline	-	-	-	-	-	-	-	-
L-alanine	+	+	+	+	+	+	+	+
L-arginine	-	-	-	-	-	-		
L-aspartic acid	-	+	+	+	+	-	+	+
L-glutamic acid	+	+	+	+	+	+	+	+
L-histidine	-	+	+	+	+	+	+	+
L-pyroglutamic acid	-	+	+	+	+	+	+	+
L-serine	-	-	-	-	-	-	-	-
Pectin	-	-	-	-	-	-	-	-
D-galacturonic acid	-	-	-	-	-	-	-	-
L-galactonic acid lactone	-	-	-	-	-	-	-	-
D-gluconic acid	-	-	-	-	-	-	-	-
D-glucuronic acid	-	-	-	-	-	-	-	-
Glucuronamide	-	-	-	-	-	-	-	-
Mucic acid	-	-	-	-	-	-	-	-
Quinic acid	-	-	-	-	-	-	-	-
D-saccharic acid	-	-	-	-	-	-	-	-
p-hydroxy-phenylacetic-acid	-	-	-	-	-	-	-	-
Methyl pyruvate	+	-	+	+	-	+	+	+
D-lactic acid methyl ester	+	-	-	-	-	-	-	-
L-lactic acid	+	+	+	+	+	+	+	+
Citric acid	+	+	+	+	+	+	+	+
α-keto-glutaric acid	+	+	+	+	+	+	+	+
D-malic acid	-	+	-	-	-	-	+	-
L-malic acid	+	+	+	+	+	+	+	+
Bromo-succinic acid	+	+	+	+	+	+	+	+

γ-amino-butyric acid	-	-	-	-	-	-	-	+
α-hydroxy-butyric acid	+	+	+	+	+	+	+	+
β-hydroxy-D,L-butyric acid	+	+	+	+	+	+	+	+
α-keto-butyric acid	+	+	+	+	+	+	+	+
Acetoacetic acid	-	-	-	-	-	-	-	-
Propionic acid	+	+	+	+	+	+	+	+
Acetic acid	+	+	+	+	+	+	+	+
Formic acid	-	-	-	-	-	-	-	-

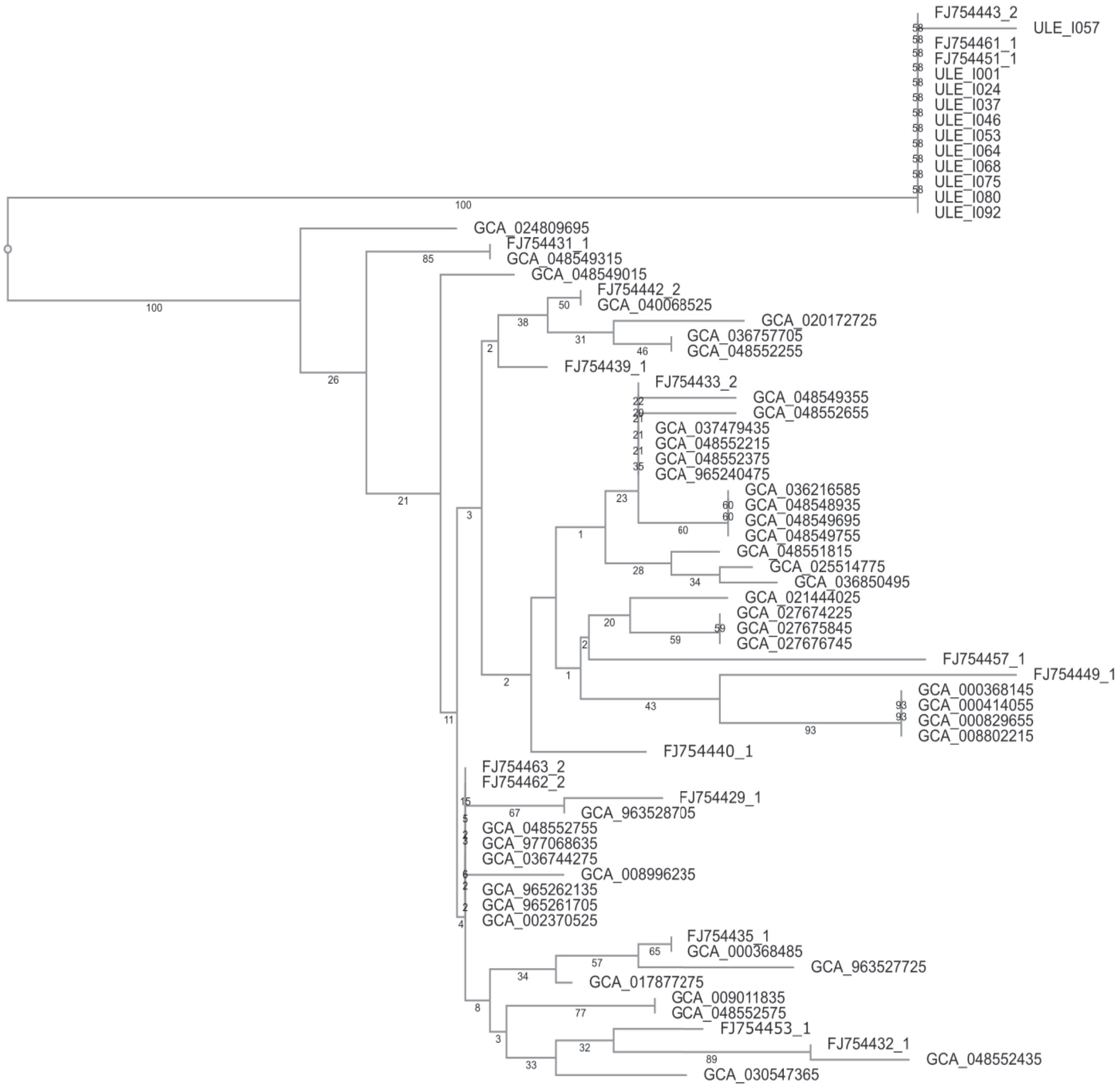

Figure S1. Partial *rpoB* gene sequence phylogenetic tree for 16 *A. guillouiae* strains from Nemec et al. 2010 (8) (FJ*), 43 *A. guillouiae* genomes from NCBI (GCA_*) and 11 *A. legionensis* sp. nov. isolates (ULE_*). FJ754443 = RUH 2236 (=LMG 10606); FJ754461 = LUH 6980; FJ754451 = LUH 5606.

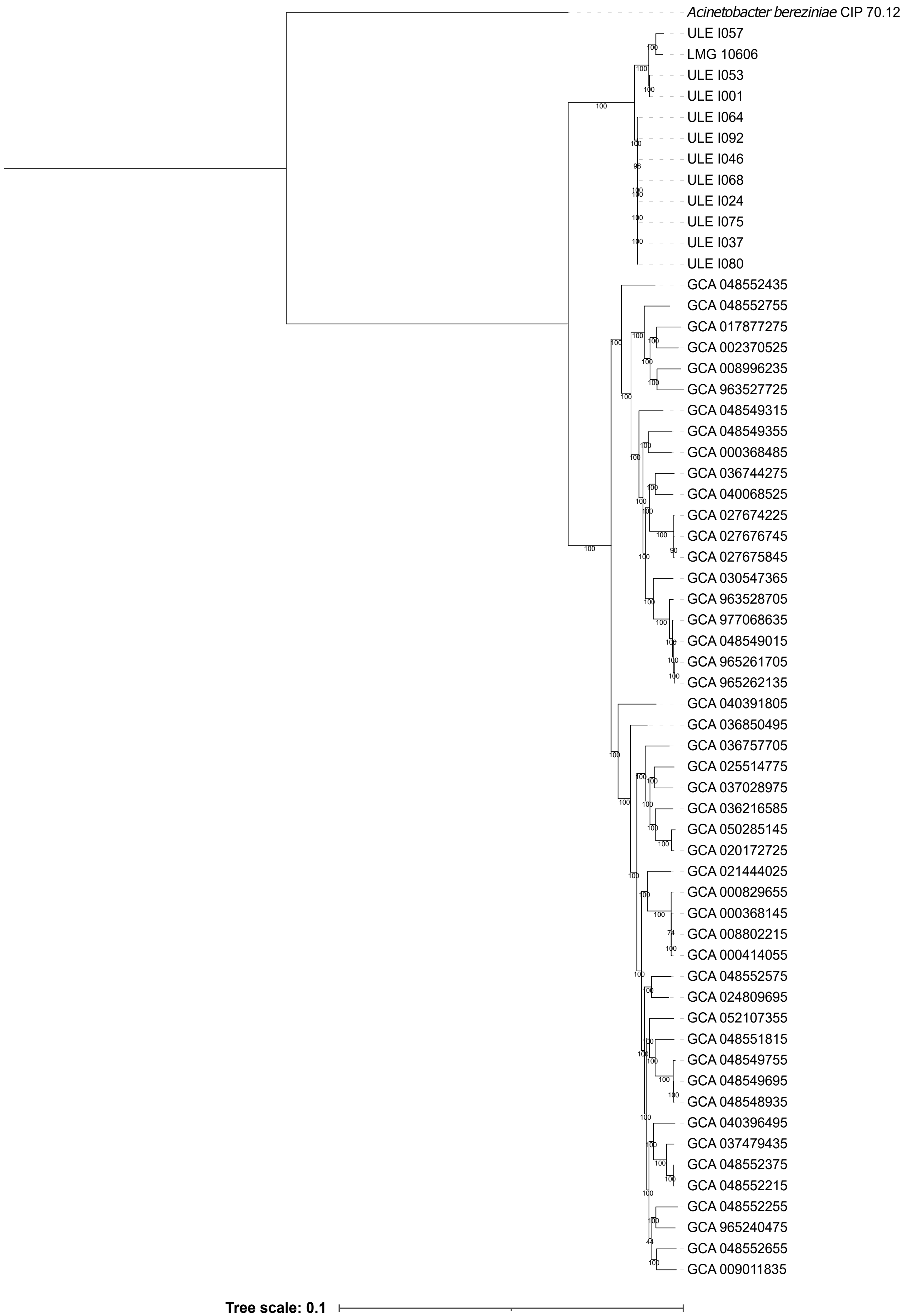

Figure S2. Core-genome tree of 48 *A. guillouiae* genomes (those with names starting by GCA), 13 *A. legionensis* genomes (names starting by ULE or LMG) and the referene genome for *A. berezinae*, as outgroup for rooting the tree.

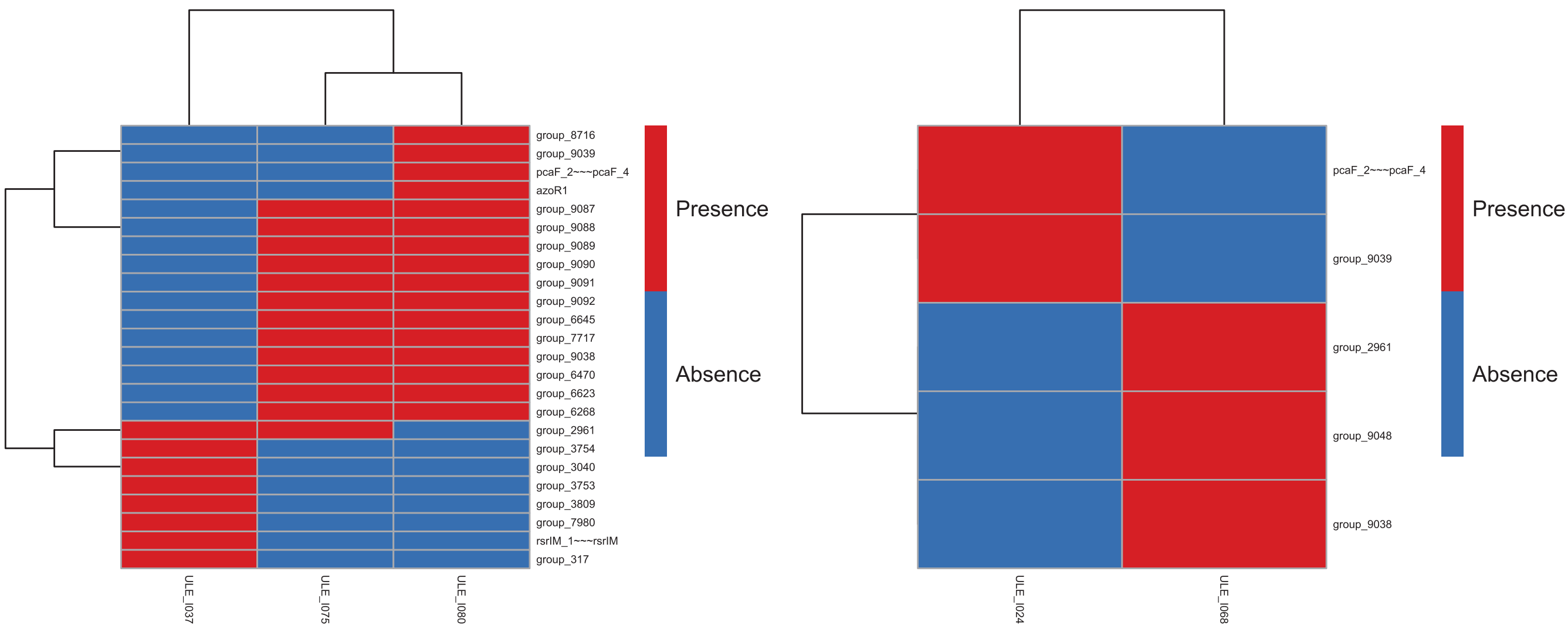

Figure S3. Accessory genes with different distributions within the 2 non-SNP different clusters found (indicated at Table S5).

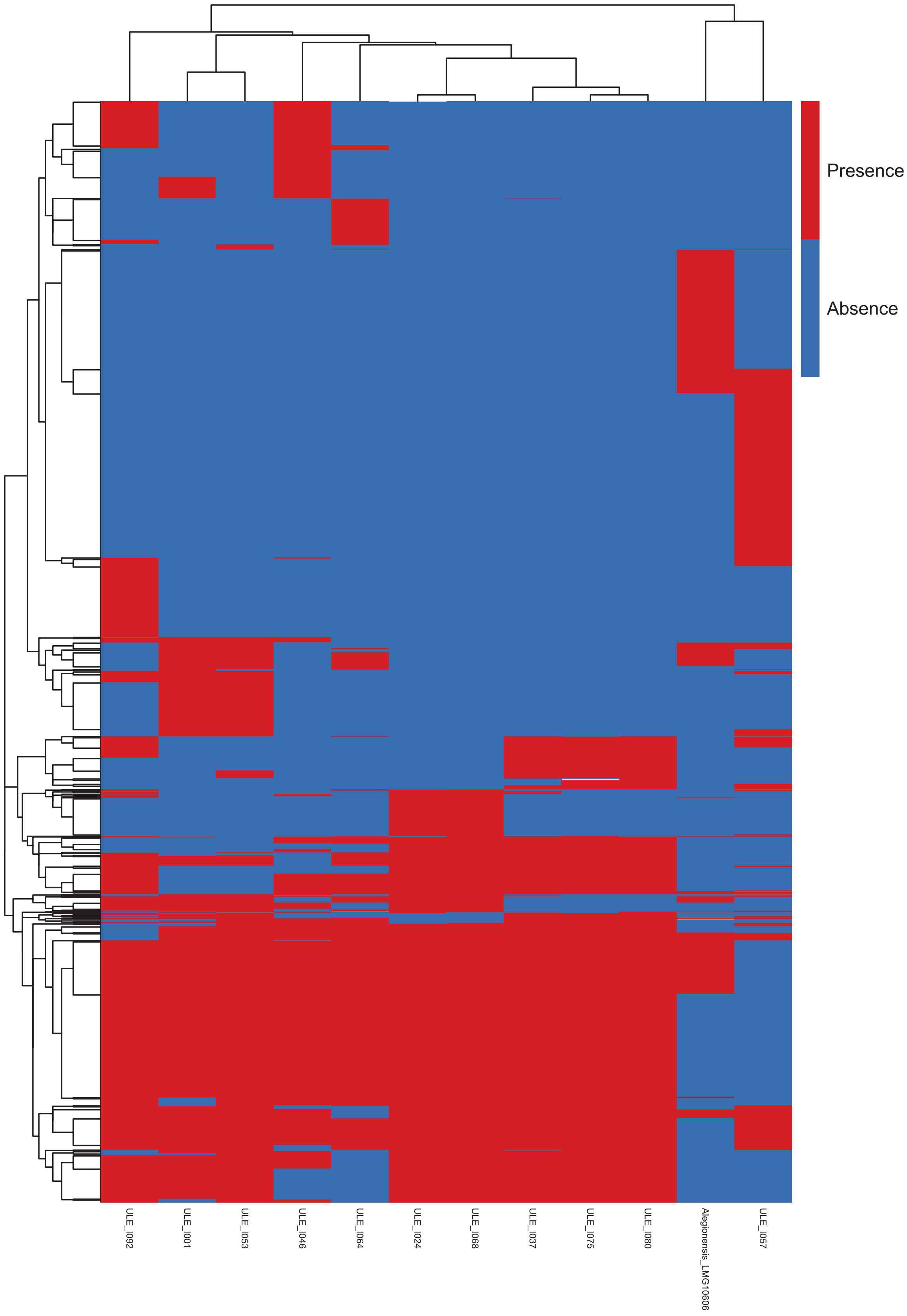

Figure S4: Accessory genes found on the 12 *A. legionensis* sp. nov. genomes.

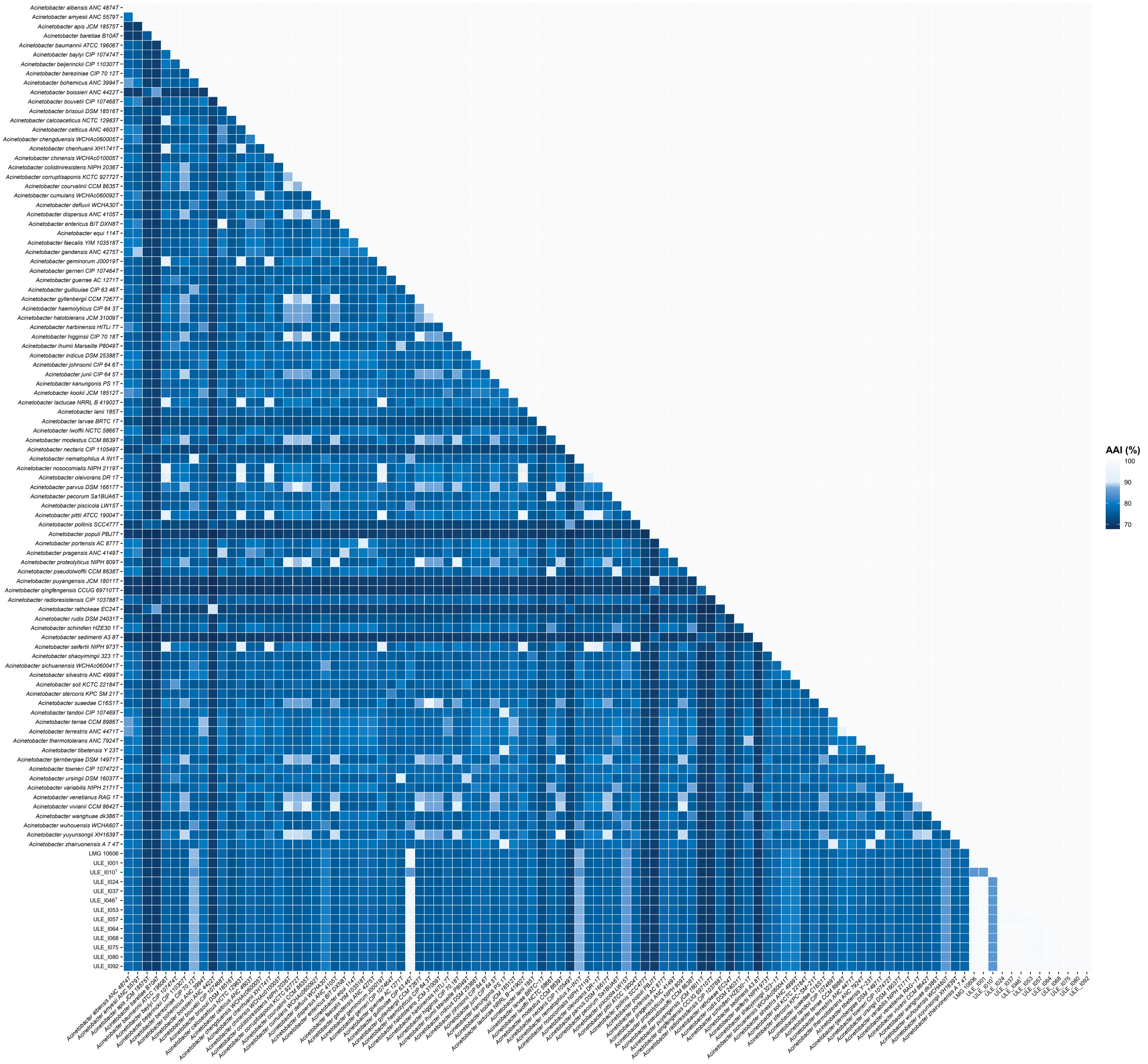

Figure S5. Average amino-acid identity (AAI) between the 12 study isolates (ULE_), strain LMG 10606, and type strains of all *Acinetobacter* species with validly published name under the ICNP.

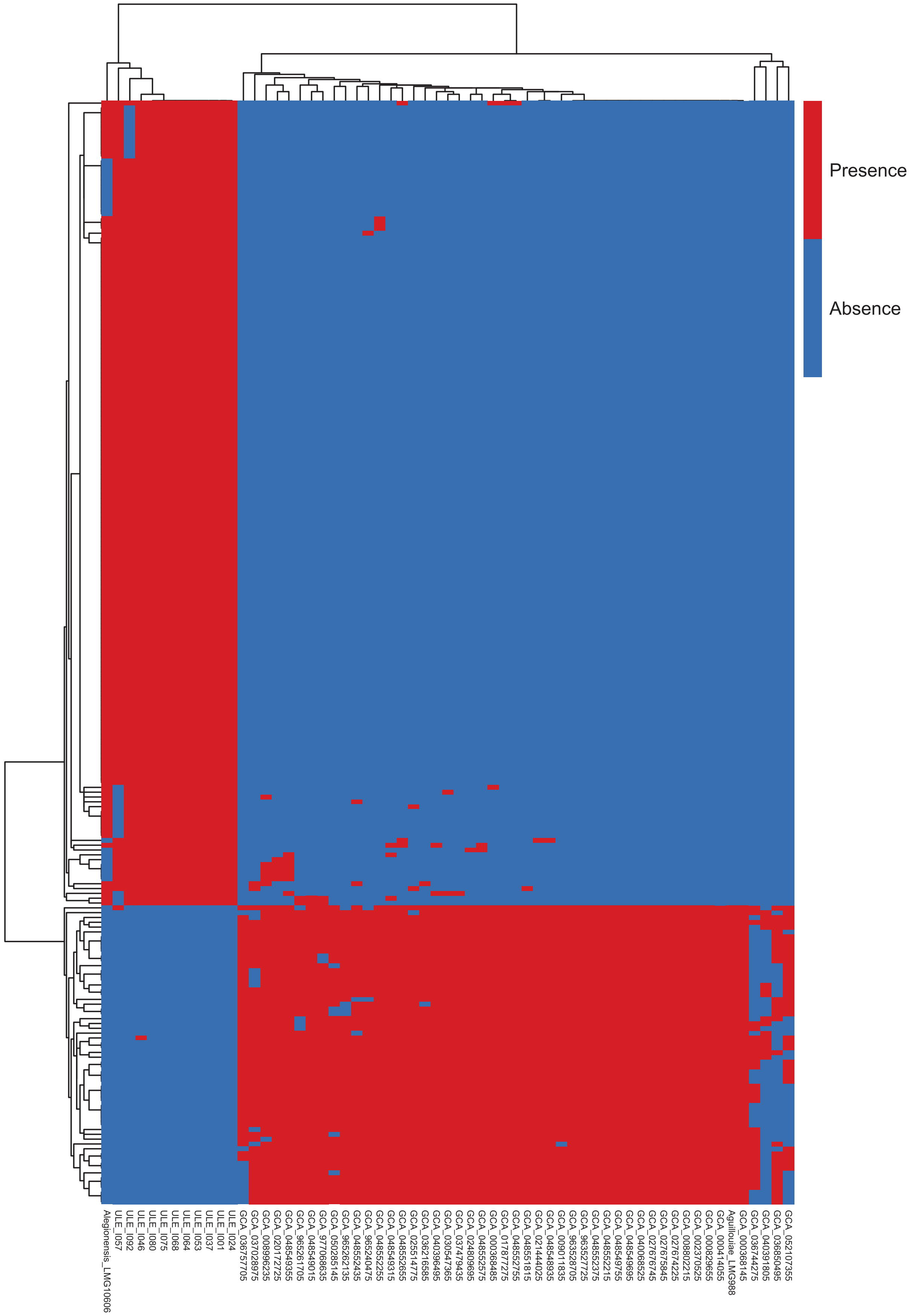

Figure S6. Accessory genes with significant prevalence between 12 *A. legionensis* sp. nov. genomes (right cluster) and 49 *A. guillouiae* genomes (left cluster).

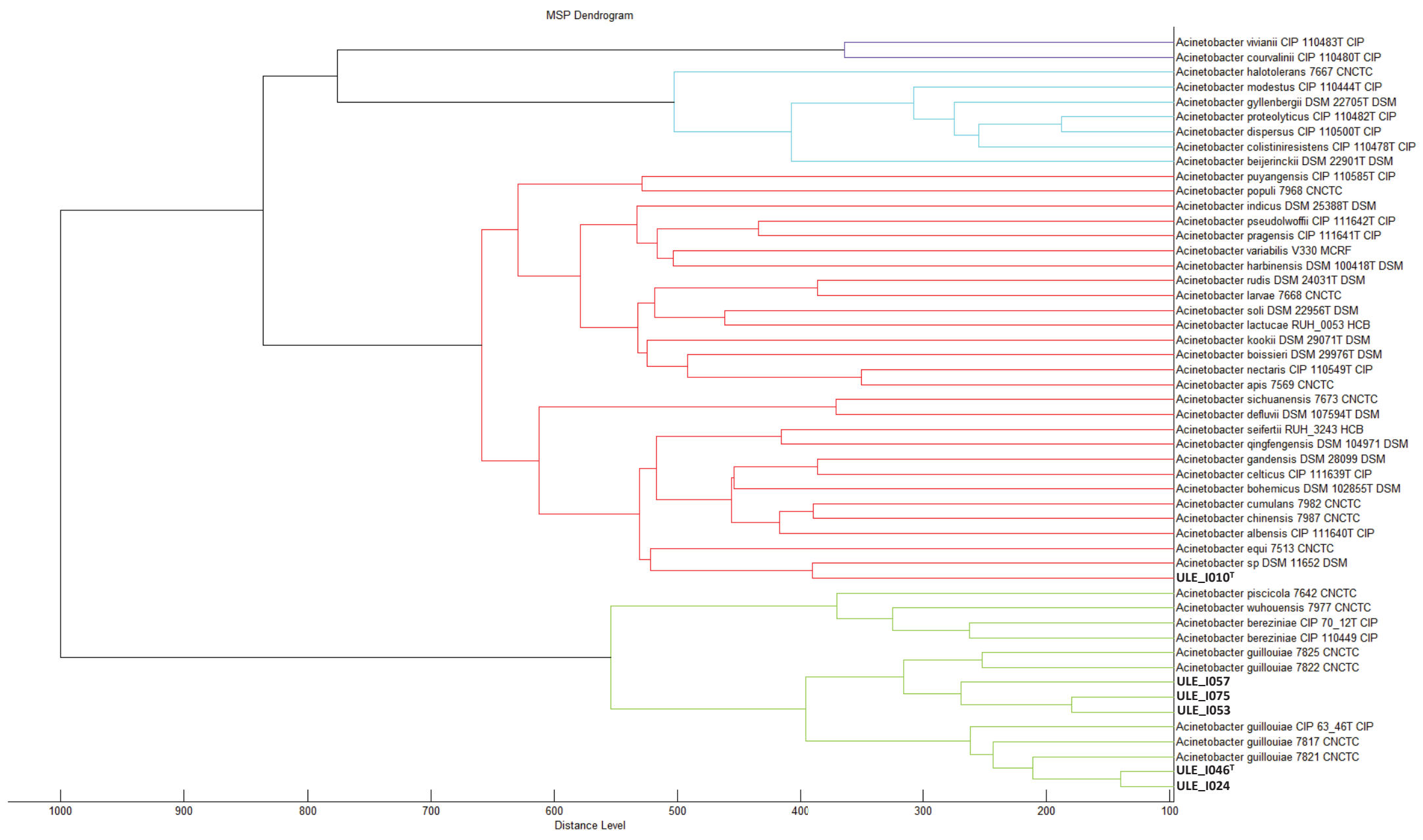

Figure S7. MSP-based dendrogram of the 6 strains selected and 45 *Acinetobacter* species from the Bruker taxonomy library.
